# Polyp-Canal Reconstruction Reveals Evolution Toward Complexity in Corals

**DOI:** 10.34133/research.0166

**Published:** 2023-06-06

**Authors:** Yixin Li, Xin Liao, Xin Wang, Yuanchao Li, Hongwei Zhao, Yunpeng Zhao, Junyuan Chen, Chunpeng He, Zuhong Lu

**Affiliations:** ^1^ State Key Laboratory of Bioelectronics, School of Biological Science and Medical Engineering, Southeast University, Nanjing 210096, China.; ^2^ State Key Laboratory of Coastal and Offshore Engineering, Dalian University of Technology, Dalian 116024, China.; ^3^ Guangxi Key Lab of Mangrove Conservation and Utilization, Guangxi Mangrove Research Center, Guangxi Academy of Sciences, Beihai 536000, China.; ^4^ Hainan Academy of Ocean and Fishery Sciences, Haikou 571126, China. ​; ^5^ State Key Laboratory of Marine Resources Utilization in South China Sea, Hainan University, Haikou 570228, China.; ^6^ Nanjing Institute of Geology and Palaeontology, Chinese Academy of Sciences, 39 East Beijing Road, Nanjing 210008, China.

## Abstract

Modern scleractinian corals are classified into robust, complex, and basal clades through comparative molecular studies. However, only few morphological or biological criteria can systematically determine the evolutionary trajectories of these major scleractinian coral clades. Here, we obtained the structural information of 21 scleractinian coral species representing robust and complex clades: High-resolution micro-computed tomography was used to reconstruct the polyp-canal systems in their colonies and to visualize the dynamic polyp growth processes. We found that the emergence of mesh-like canals may distinguish representatives of complex and robust clades. The differences in polyp-canal connections suggest distinct evolutionary trajectories among coral species: The formation of the canal network promoted the development of more complex coral structures, and coral polyps within this network formed calices of very similar volume, following precise axial growth directions. The influence of individual polyps on the coral colony becomes less significant as coral structures become more complex, and coral species with more complicated polyp-canal systems occupied niches more efficiently. This work supplements current evolutionary studies on reef-building corals, providing insight for further studies on coral growth patterns.

## Introduction

Coral reefs are the most diverse marine ecosystems and are supported by the continued healthy growth of reef-building scleractinian corals [1]. Multiple aspects of biological research on reef-building corals can shed light on their ecological, evolutionary, and economic significance [2]. According to comparative molecular studies, modern scleractinian corals are classified into robust, complex [3], and basal [4] clades, and this nomenclature is supported by most follow-up multi-omics studies [5–13] (Table). However, only few existing morphological or biological criteria can systematically determine the evolutionary trajectories of robust and complex clades. The interactions and connections among polyps in the same colony are important during coral growth [14]. Unfortunately, these factors are usually ignored when we only focus on differential gene expression patterns, which limits studies on the evolution of biological complexity in corals [15,16]. The construction of evolutionary trees is further challenged by the evolutionary mechanisms of reef-building corals, which include incomplete lineage sorting of shared ancestral polymorphisms and deep divergence [17,18]. Although existing morphological studies provide intuitive structural information of reef-building corals to complement systematic molecular studies [19], they are limited by their methods and techniques as they fail to obtain several aspects of growth information that are hidden in the opaque skeletons [20].

**Table.** Systematic molecular studies supporting the evolution of modern scleractinian coral clades.

Method	Achievement	Value
Mitochondrial 16*S* ribosomal gene sequences	Reef-building corals are classified into robust and complex clades.	The great morphological diversity of current scleractinians can reflect the origins of coral skeletons.
EST sequencing	Both BMP and Wnt pathways are involved in the osteogenesis of robust coral *S. pistillata*	Extending our understanding of “robust” corals from the view of physiological processes.
Molecular and morphological analyses	Modern shallow-water corals had multiple independent origins from deep-water ancestors.	Providing new insights into the origins and diversification of Scleractinia corals.
Genomics and transcriptomics	Analyzed the data of intracellular photosynthetic dinoflagellate symbionts in robust and complex corals.	Elucidating the evolutionary strategies among coral species.
Genome assembly	A fungal-like histidine biosynthesis pathway is present in robust corals but not complex corals.	Robust corals can synthesize histidine de novo.
Embryo tissue sections	Complex corals have a well-developed blastocoel and no prawn chip stage.	Beneficial for understanding the early developmental patterns and functional implications during coral growth.
Embryo tissue sections	Morphological characteristics during embryogenesis facilitate the grouping of robust and complex coral clades.	Clarifying a deep division in the Scleractinia.

The rapid development of high-resolution micro-computed tomography (HRCT) facilitates the acquisition of structural information [21–23]. In this study, 3-dimensional (3D) reconstructions of internal canals provided a tool for determining growth information buried within coral skeletons, which could fill the knowledge gaps in current evolutionary coral studies [14,21,24,25]. Using HRCT, we obtained the morphological information of representative scleractinian reef-building corals from 9 families, 14 genera, and 21 species in the Indo-Pacific region and reconstructed the canals and lumen in calices of each colony to visualize their polyp-canal connections during coral growth. The polyp-canal relations revealed the evolutionary trajectories among these coral species and influenced the coral distribution in different habitats. Furthermore, to obtain information on dynamic coral growth, we also visualized the polyp growth and budding processes and calculated the luminal volume in compound coral calices. These studies revealed the impact of the canal network on regulating synergistic growth and enhancing growth efficiency.

This work characterizes the morphological differences between robust and complex coral clades and the importance of polyp-canal connections in coral evolution. Our studies and the existing systematic molecular studies complement each other to provide insights into the profoundly complex coral growth patterns that dictate coral evolution.

## Results

### Five polyp-canal types in coral species

**Fig. 1.**
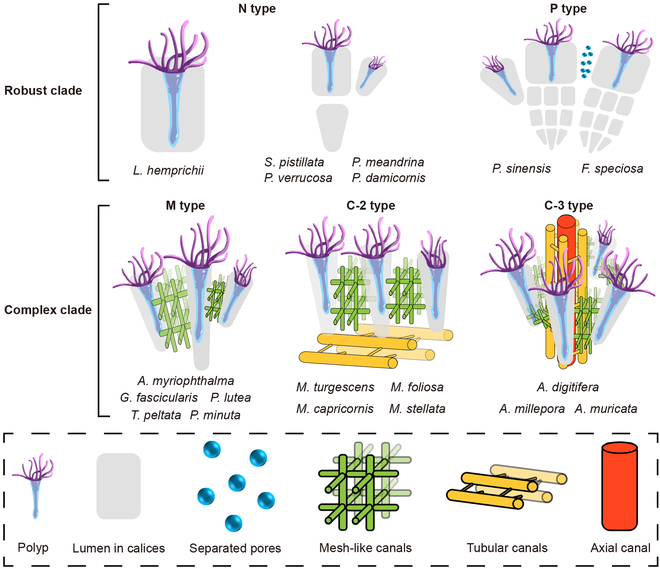
Five structural types of polyp-canal systems in compound coral species. The robust coral clade contains 2 types of polyp-canal systems: N type and P type. There are no canals connecting polyps in N type colonies, while separated pores are found among polyps in P type colonies. Mesh-like canals connect all polyps in colonies of complex coral clades. The system in the M type contains only mesh-like canals, while tubular canals are found in the canal network in the C-2 type. The most complex system is formed with mesh-like canals, tubular canals, and the axial canal and is called the C-3 type.

We reconstructed the polyps and canal systems of 20 reef-building corals through HRCT analysis. There are 4 canal forms in the coenosteum and corallite walls between adjacent polyps: separated pores, mesh-like canals, tubular canals, and the axial canal. We found that these 4 canal forms were observed in 5 polyp-canal system types in compound coral colonies (Fig. 1), which led to the classification of robust and complex coral clades, and the evolution of these species.

In the robust clade, the only canal form found in the coenosteum was the separated pores. There are no canals among adjacent polyps of *Lobophyllia hemprichii*, *Pocillopora damicornis*, *Pocillopora meandrina, Pocillopora verrucosa*, and *Stylophora pistillata*, so they belong to the “No-canal type” (N type). There are some separated pores in the coenosteum between the calices of *Favia speciosa* and *Platygyra sinensis*, which we named “Pores-between-polyps type” (P type). The emergence of mesh-like canals was the morphological criterion that distinguished robust and complex clades, and they were common among all polyps in complex coral colonies. In *Galaxea fascicularis*, *Pavona minuta*, *Turbinaria peltata*, *Porites lutea*, and *Astreopora myriophthalma*, the polyp-canal systems belong to the “Mesh-like canals type” (M type), which can be considered the original form of the canal network with only one canal form. In our 4 *Montipora* coral species (*Montipora turgescens*, *Montipora capricornis*, *Montipora foliosa*, and *Montipora stellata*), the canal networks are constituted of both mesh-like canals and tubular canals, and we designated this “Canal network with two canal types” (C-2 type). Consequently, the more complex and fine system in the *Acropora* species (*Acropora digitifera*, *Acropora millepora*, and *Acropora muricata*) with 3 canal forms (mesh-like canals, tubular canals, and axial canal) was called “Canal network with three canal types” (C-3 type).

As for the solitary corals *Cycloseris vaughani* and *Balanophyllia europaea*, multiple canals can be found in their mouth, below the only polyp in their colonies.

### Growth patterns and processes of robust corals

Among the robust corals, *C. vaughani* is a solitary large-polyp scleractinian with a large lumen in the mouth area for its polyp (about 10 mm × 8 mm × 5 mm in 40 mm × 36 mm × 10 mm sample) (Fig. S1). In this monostomatous corallum, there are multiple canals in the porous skeletons below the coral polyp, which is conducive to its early attachment and growth. Since there is only one polyp in *C. vaughani*, there is no need for a canal system connecting adjacent polyps in a colony. Therefore, its skeleton formation is relatively simple, containing only some septa with spiny septal margins that radiate from the center to the outer edge of the corallum, and the undersurface skeletons below the polyp.

*L. hemprichii*, a compound large-polyp scleractinian, is similar to a combination of multiple *C. vaughani* in one flabello-meandroid colony (Fig. S2). The calices are tightly connected with various septas inside, with no canal system among adjacent calices (Fig. S2A). Polyps in one colony form calices with irregular lobes, resulting in diverse structures, while calices belonging to adjacent polyps with a budding relationship are joined at the base (Fig. S2B).

**Fig. 2.**
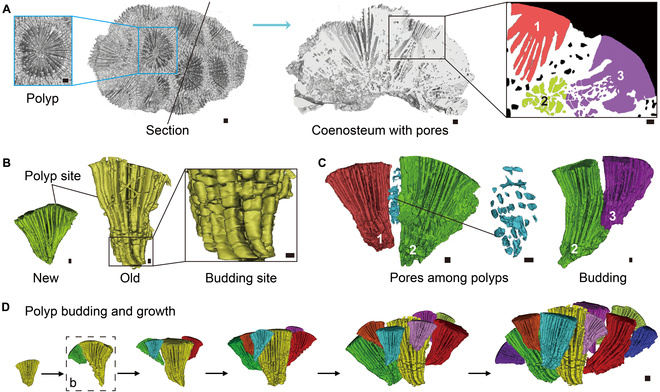
The polyp-canal system and colony-forming process of robust coral *F. speciosa*. (A) Reconstructions of *F. speciosa* colony. The “1,” “2,” and “3” labels denote polyp sample nos. 1 to 3. (B and C) 3D reconstructions of the polyp-canal system in *F. speciosa*. (D) Polyp budding and growth process during colony formation. Scale bars: (A) 1 mm; (B) 0.5 mm; (C) 0.2 mm; (D) 1 mm.

In the compound large-polyp scleractinians, *F. speciosa* and *P. sinensis*, the lumens in the coral calices can be divided into 2 regions with significant morphological differences: the polyp site and the budding site (Fig. 2 and Fig. S3). In *F. speciosa*, the upper part of the inverted cone-shaped lumen in each coral calyx is the polyp site, which is similar to the one in *C. vaughani* (Fig. 2B). The lower part of the lumen is formed with multiple vertically arranged cubes, which are separated by the columella, septum, and dissepiment. As this part of the lumen connects the lumens of newly budded polyps, we call it the budding site. No budding site is present in the calices of newly budded polyps due to the lack of dissepiments between neighboring septa. Separated pores exist inside the coenosteum among polyps (Fig. 2C). There are no pores in the skeleton between polyps with budding relations, while several pores exist in the skeletons between the polyp sites of adjacent polyps without budding relations. The polyps in *F. speciosa* colonies follow a cluster form growth and budding pattern (Fig. 2D). As another species with the P type polyp-canal system, *P. sinensis* has similar growth patterns as *F. speciosa*; the main difference between them is that the lumens in the *P. sinensis* calices are relatively slender (Fig. S3).

As compound small-polyp scleractinians, there are no pores or canals in the coenosteum of our 4 *Pocillopora* and *Stylophora* species. The inter-septal spaces show a deterministic growth form and are the only lumens in these coral colonies, which have an N type polyp-canal system (Fig. S4). The inter-septal spaces are separated by nonporous walls and dissepiments, and the spaces belonging to one polyp form a turbinate-shaped lumen in its calyx (Fig. S4B). The first inter-septal space belonging to the newly budded polyp is similar to an inverted cone, while the following ones are like cubes. The polyp site is the top space of each calyx (Fig. S4C). Although the polyps in these *Pocillopora* and *Stylophora* corals exhibit axial growth [14], their polyp growth and budding pattern are also of the cluster form, just like other large, robust corals (Fig. S4D).

### The evolution of canal systems and growth direction in complex corals

In the complex coral clade, *B. europaea* is a solitary large-polyp scleractinian with similar skeletal structure with *C. vaughani* (Figs. S1 and S5). Porous skeletons for early attachment can be found below the polyp site, and multiple canals are formed inside these skeletons.

In the compound large-polyp scleractinian *G. fascicularis*, polyp and budding sites are clearly distinguished in the calices, similar to those in *F. speciosa* and *P. sinensis* (Fig. 2 and Figs. S3 and S6B). The corallites in *G. fascicularis* are 2 to 3 mm apart, a distance that is longer than that of robust corals (Fig. S6A). The coenosteum between corallites has a structure similar to graphene sheets, with the original mesh-like canals inside. The canals in the coenosteum near the bottom of the corallites are more distinct, connecting polyps with budding relations (Fig. S6). In *G. fascicularis* colonies, polyp growth is relatively asynchronous, and the slower-formed calices become sealed by the coenosteum, resulting in the death of the polyps and the filling of the inner space by mineralizing bacteria (Fig. S6C).

*P. minuta* is a compound large-polyp scleractinian whose colony encrusts rubble or sediment in coral reefs like a thin wrapper (Fig. S7). Visible lines can be found on the septa among adjacent immersed calices, linking all polyps in the colony (Fig. S7A). Mesh-like canals can be found among the top of polyps, and all these canals form a large net, connecting all polyps in the colony to gather them together (Fig. S7B). However, these polyps still have low consistency in forming calices; thus, the lumen in their calices have different shapes and sizes (Fig. S7C).

*T. peltata* is another compound large-polyp scleractinian (Fig. S8). The structure of the lumen in calices is similar to *G. fascicularis*. However, instead of those graphene-sheet-like skeletons, porous coenosteum with mesh-like canals fills up the area among calices, wrapping all polyps with or without budding relationship (Figs. S5 and S7B). Polyps in a colony are widely spaced and expand like blooming petals (Fig. S8C).

**Fig. 3.**
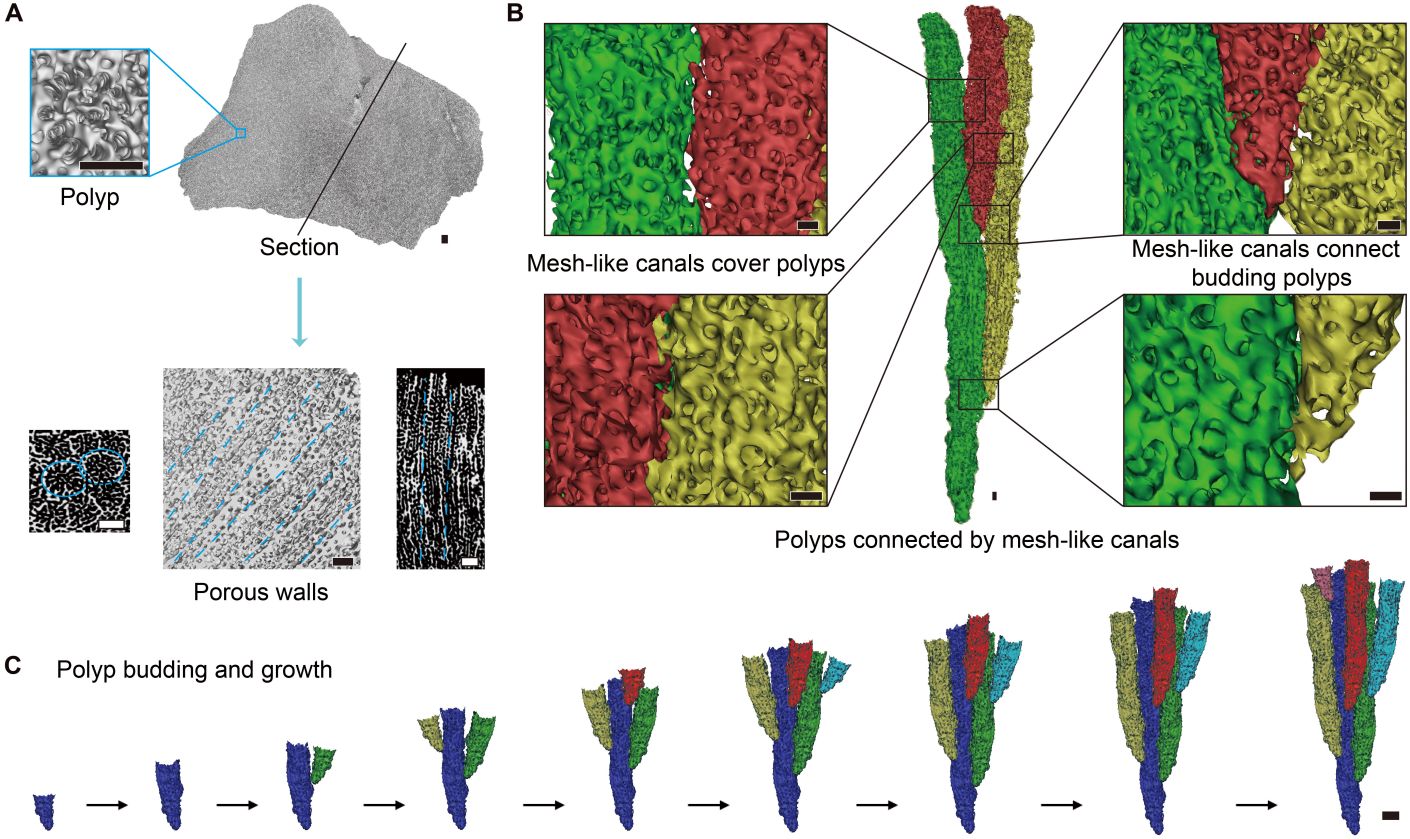
Structure of the complex coral *P. lutea* with mesh-like canals. (A) Reconstructions of the *P. lutea* colony. (B) 3D reconstructions of the polyp-canal system in *P. lutea*. (C) Polyp budding and growth process during colony formation. Scale bars: (A) 1 mm; (B) 0.2 mm; (C) 1 mm.

*P. lutea* is a compound small-polyp scleractinian. Its colonies are filled with skeletal elements, and its corallites are challenging to discern. However, our reconstructions of the polyp-canal system can clearly reveal the formation of the coral colony (Fig. 3). As the coenosteum is scarce in *P. lutea*, the thin layer of mesh-like canals is formed in the wall of closely packed corallites, covering and connecting all polyps in the colony (Fig. 3B).

In the encrusting compound small-polyp scleractinian *A. myriophthalma*, the skeleton is porous with a reticulate coenosteum, while the calices are distinct and separate (Fig. S9). The polyps are far apart from each other and connected by mesh-like canals in the coenosteum and corallite walls; these canals occupy a larger space in *A. myriophthalma* than *P. lutea* (Fig. 3B and Fig. S9B). The polyp growth and budding patterns in the 3 species with M type polyp-canal systems are similar (Fig. 3C and Figs. S6D and Fig. S9C).

**Fig. 4.**
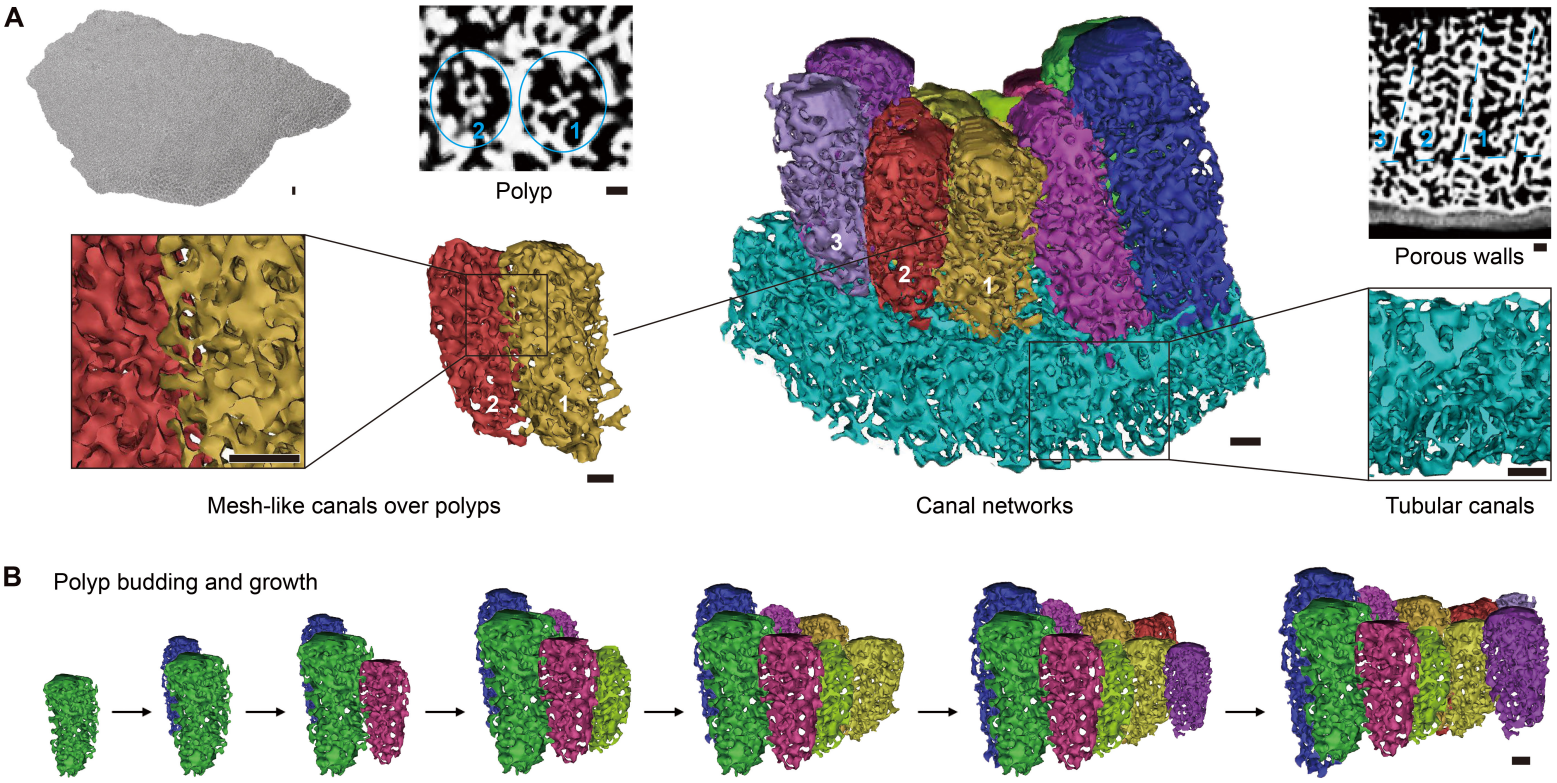
Growth patterns and the canal network in complex coral *M. turgescens*. (A) 3D reconstructions reveal polyp-canal connections in *M. turgescens*. The “1,” “2,” and “3” labels denote polyp sample nos. 1 to 3. (B) The polyps form laterally continuous platy colonies under the control of the canal network. Scale bar: 0.2 mm.

The skeleton formation of corallites in the compound small-polyp scleractinian *M. turgescens* is difficult to distinguish from that of *P. lutea*, and there are also mesh-like canals covering all polyps to connect neighboring calices (Figs. 3B and 4A). The most obvious difference in *M. turgescens* relative to *P. lutea* is the canal structure beneath the calices. The *Montipora* corals form horizontal skeletal rods near the colony base, enclosing the horizontal tubular canals below the polyps and connecting all calices in one colony (Fig. 4A). Thus, the mesh-like canals and tubular canals have different distributions and functions, giving rise to the C-2 type polyp-canal system that regulates the growth process of all polyps in one colony. This system ensures more refined signal transmission and transport of nutrients, secretions, and calcifications among polyps, leading to better regulation of coral growth and budding directions to form laterally continuous platy colonies (Fig. 4B).

In *M. foliosa* and *M. capricornis* colonies, the distance between neighboring calices is larger than in *M. turgescens* (Fig. 4A and Fig. S10A). Mesh-like canals among polyps are parallel to the direction of calyx formation and are enclosed by a network of long, prominent rods and short lateral bars (Fig. S10A). Tubular canals are formed along the growth direction of the coral colony beneath all polyps. The polyps’ horizontal tile growth and budding process result in the formation of laminar whorl-like colonies (Fig. S10B).

*M. stellata* is a coral species with both horizontal and vertical growth patterns, and its polyp-canal system is reminiscent of a transitional evolutionary state (Fig. S11). The tubular canals are concentrated in the columnar area at the center of the coral branches, just like the original form of the axial canal in the axial corallite. The structure and distribution of mesh-like canals in its C-2 type system are similar to those in *M. foliosa* (Figs. S10A and S11A). The *M. stellata* corals exhibit axial growth and budding patterns: Coral branches with lateral growth axes extend horizontally, and coral branches with longitudinal growth axes extend vertically (Fig. S11B).

**Fig. 5.**
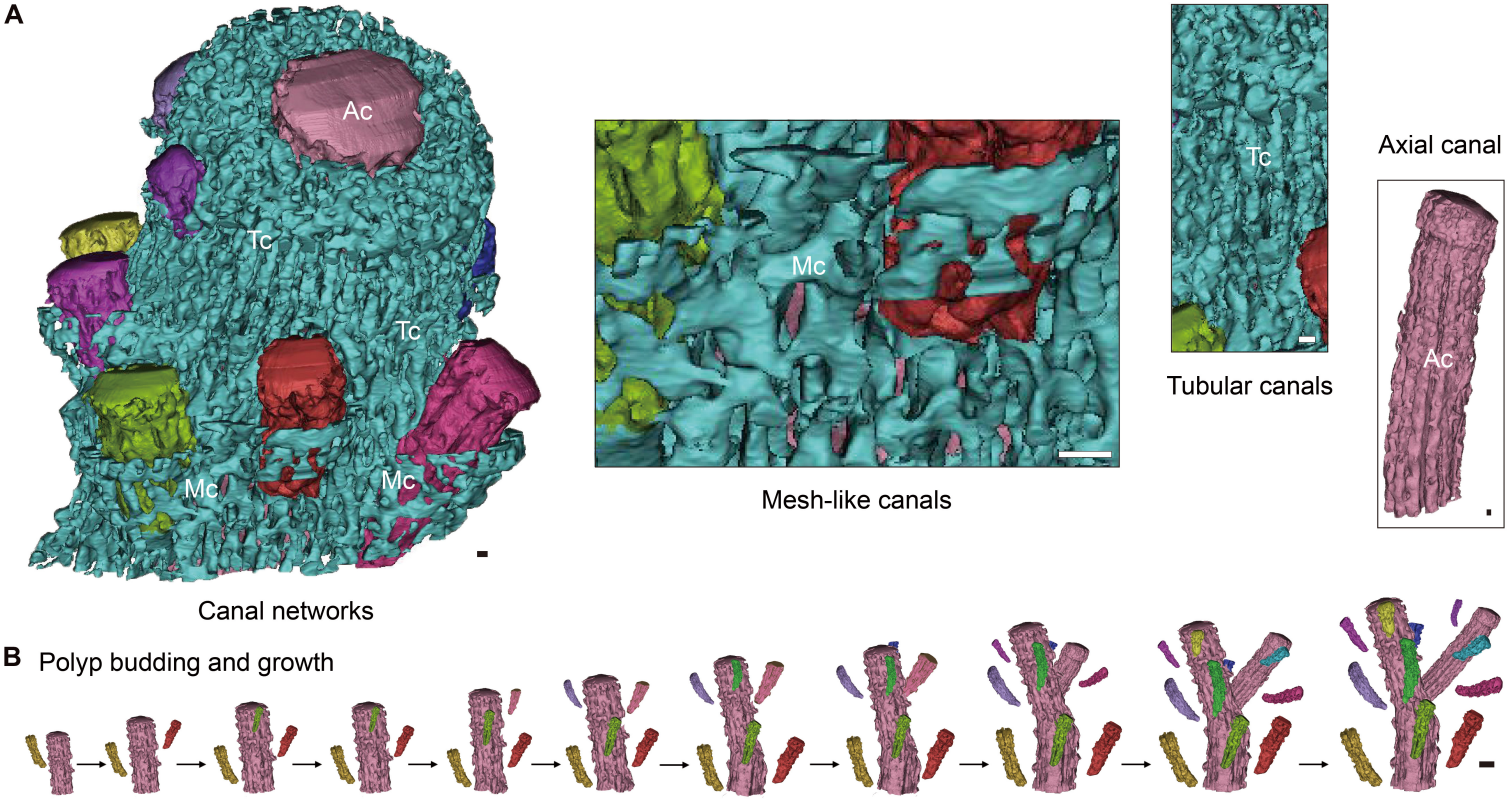
Growth patterns and canal networks in 3 *Acropora* coral species. (A) 3D reconstructions reveal polyp-canal connections in the 3 *Acropora* species (Figs. S12 to S14). The mark “Mc” means mesh-like canals, “Tc” means tubular canals, and “Ac” means axial canal. (B) The polyps follow axial growth directions and form vertical dendroid growth colonies. Scale bars: (A) 0.1 mm; (B) 0.5 mm.

The 3 *Acropora* species in this work are dendroid compound small-polyp scleractinians with the most complex C-3 type polyp-canal system (Fig. 5). The structures of the polyp-canal systems in these species are similar; the only difference is the canal network coverage over the newly budded polyps at the branch tip. The lowest coverage is observed in *A. millepora*, followed by *A. muricata* and then *A. digitifera* (Figs. S12 to S14). The axial canals in these 3 *Acropora* species dictate the directions of the polyp growth and canal system formation, while deterministic canals are formed along the axial canals (Fig. 5A). Neat, regular mesh-like canals are formed in the radial corallites, including in the coenosteum and walls. Tubular canals parallel to the direction of growth are formed in axial corallites surrounding the axial canal. This canal network, comprising 3 canal types, is the most complex canal system in common reef-building corals. The polyps in these *Acropora* corals follow axial growth directions and form dendroid vertical growth colonies (Fig. 5B).

### Polyp-canal connections reveal coral evolutionary trajectories

**Fig. 6.**
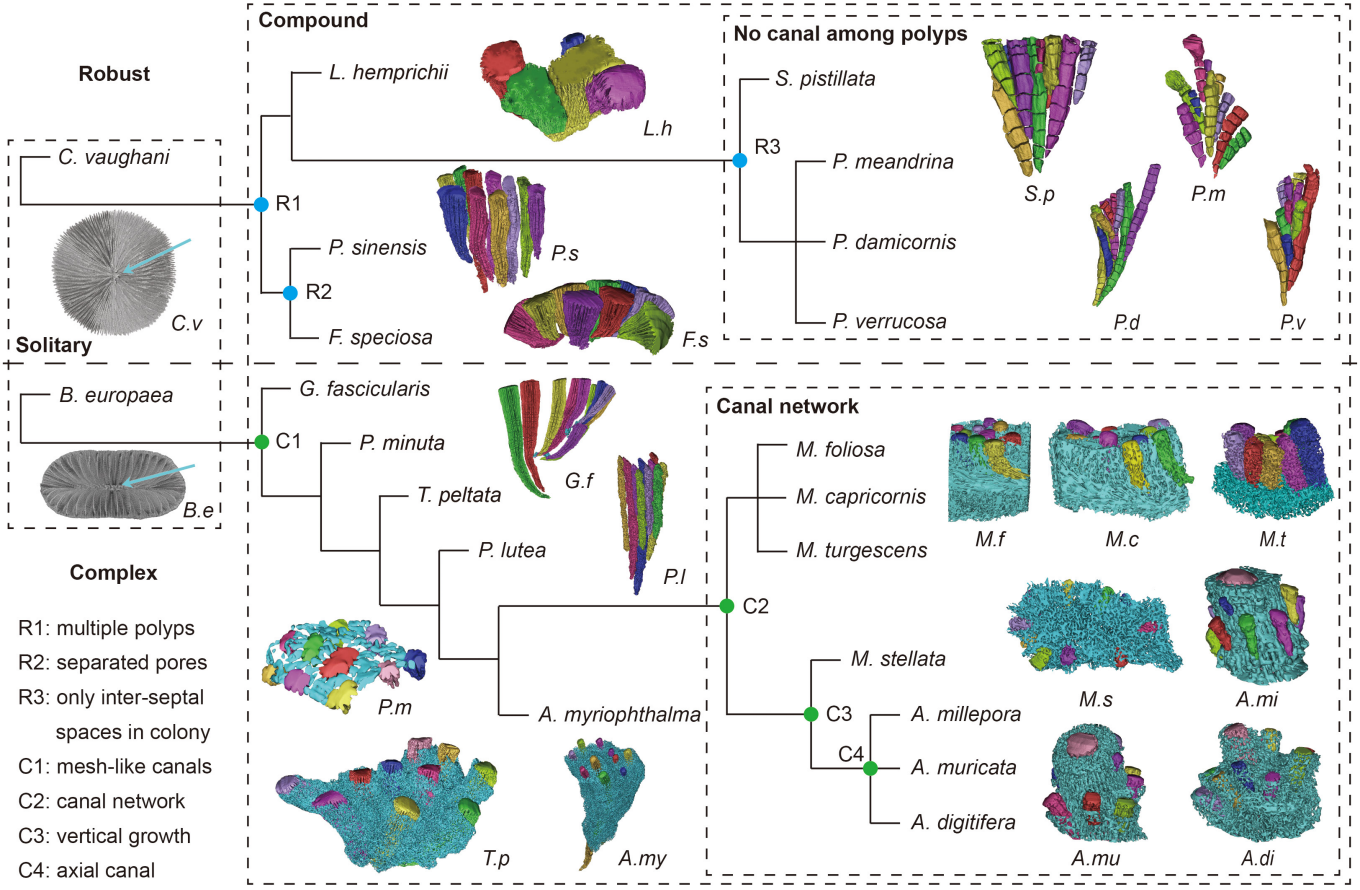
The evolutionary trajectories of polyp-canal connections in robust and complex coral clades. There are 3 main steps during the evolution of polyp-canal systems in robust corals, including “R1” (from solitary coral to compound coral), “R2” (the existence of separated pores among polyps), and “R3” (the ordered network of inter-septal spaces). There are 4 main steps in the evolution of complex coral clades: “C1” (the existence of mesh-like canals), “C2” (the formation of canal networks), “C3” (the survival strategy of vertical growth), and “C4” (the existence of the axial canal).

These 3D reconstructions successfully describe important coral growth patterns, such as the different types of polyp-canal systems, the polyp budding process, and colony formation, which provides insight into the evolution of robust versus complex reef-building coral clades. Thus, we visualized the evolutionary trajectories of robust and complex coral species based on polyp-canal connections and colony-forming processes (Fig. 6). In the robust clade, there are 3 main steps during the evolution of polyp-canal connections. First, multiple polyps exist in coral colonies after the speciation of the solitary coral *C. vaughani*. Then, in *F. speciosa* and *P. sinensis*, separated pores are found among polyps without budding relations. Meanwhile, there are still no canals among the polyps of *L. hemprichii*. As for the other 4 coral species with N type polyp-canal systems, the inter-septal spaces of *S. pistillata*, *P. meandrina*, *P. damicornis*, and *P. verrucosa*, which contain coral polyps and record their growth trajectories, are the only kind of lumen in these colonies. For complex clades, 4 main criteria are found during the evolutionary process. First, after the speciation from solitary corals like *B. europaea* to compound corals, mesh-like canals connect all polyps in the colonies of *G. fascicularis*, *P. minuta*, *T. peltata*, *P. lutea*, and *A. myriophthalma*. Then, canal networks consisting of 2 canal forms are found in *M. turgescens*, *M. capricornis*, *M. foliosa*, and *M. stellata*. Meanwhile, tubular canals gather in the center of *M. stellata* branches, resembling the axial canal’s original form, leading to the coral colony’s vertical growth. Finally, the axial canal exists in the canal network of *A. digitifera*, *A. muricata*, and *A. millepora*, forming the most precise C-3 type canal network.

### Polyps tend to be similar in corals with complex canal networks

The 3D reconstructions reveal that, in small polyp scleractinian samples, the polyps in each colony form calices with almost the same shape. However, only the *Acropora* and *Montipora* coral species form calices with similar sizes within one colony. To study the changes in the space occupied by coral polyps in each colony, we selected 3 replicate samples of each species (except *C. vaughani*, *L. hemprichii*, and *B. europaea*) and randomly selected 20 calices in each sample. To avoid the impact of newly formed calices on the results, we avoided the coral branch tips while choosing calices. We reconstructed the lumens in these selected calices and calculated their volumes (Fig. S15).

**Fig. 7.**
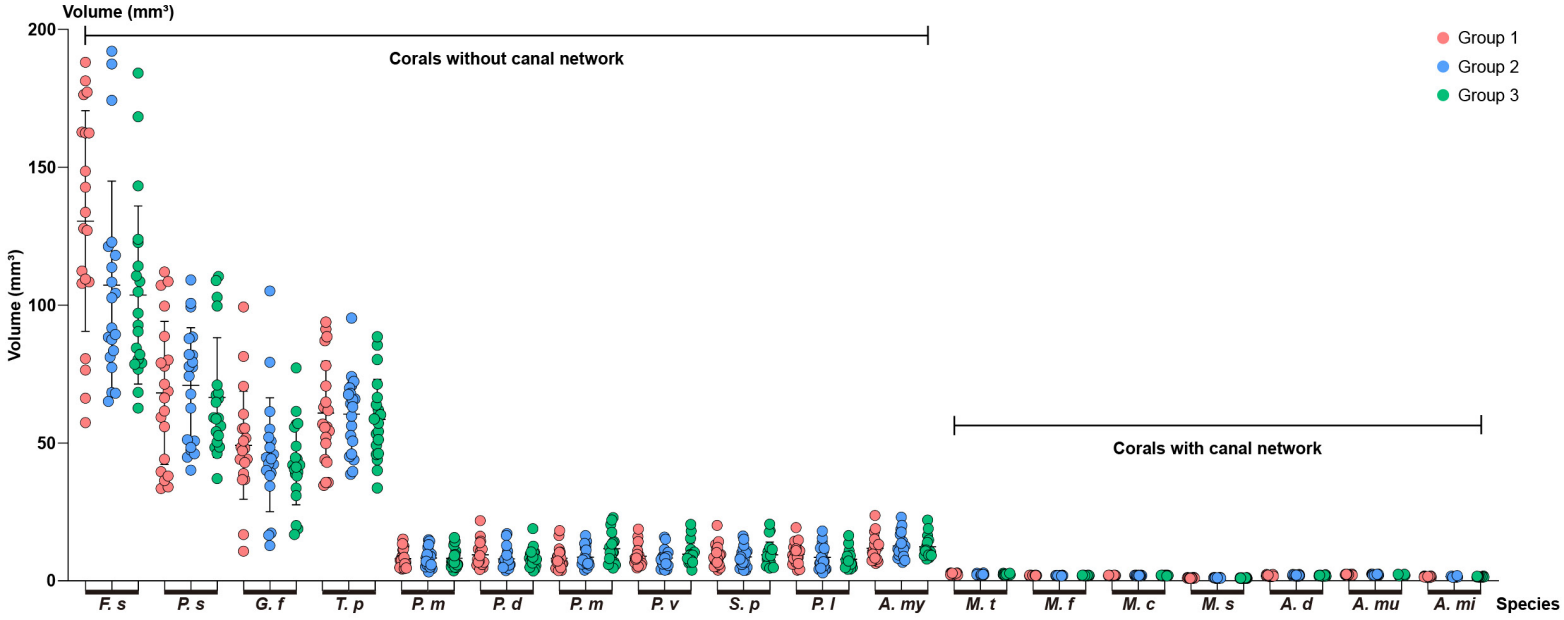
Formation patterns of calices in compound corals. We calculated the volume of the lumens in compound coral calices (except *L. hemprichil*a). Three replicate samples were analyzed for each species, and we measured 20 lumens in each sample (Fig. S15 and Table S1). The standard deviation is higher in the measurements of corals without canal networks and lower in corals with canal networks.

The results showed that among the large-polyp scleractinians, *F. speciosa*, *P. sinensis*, *G. fascicularis*, and *T. peltata*, the size of polyps was extremely discrete, and the standard deviation of each group was between 13 and 40 (Fig. 7 and Table S1). The polyp size of *P. minuta* was also discrete, with a standard deviation around 3, lower than the other 4 large-polyp scleractinians due to their smaller polyps. Among them, the standard deviations of robust corals (*F. speciosa* and *P. sinensis*) were larger, indicating relatively more considerable volume differences among polyps. Among the other small-polyp scleractinians, the dispersion of samples in the colonies without canal networks (*P. damicornis*, *P. meandrina*, *P. verrucosa*, *S. pistillata*, *P. lutea*, and *A. myriophthalma*) was relatively minimal, with standard deviations between 3 and 5.5. The sizes of the 3D reconstructions for the colonies with canal networks (*A. millepora*, *A. muricata*, *A. digitifera*, *M. capricornis*, *M. foliosa*, *M. stellata*, and *M. turgescens*) were also quite similar, with standard deviations below 0.4. Therefore, polyps form calices of similar size in coral colonies with canal networks. Once the lumens in the calices reach the desired size, the polyps almost stop forming their own calices. In coral colonies without canal networks, polyps constantly form larger calices during coral growth.

## Discussion

### The emergence of mesh-like canals accelerates the complex evolution of coral structures

The 3D reconstructions in this work demonstrate that the differences among polyp-canal systems illustrate the evolutionary trajectories in robust and complex coral clades (Figs. 1 and 6). Mesh-like canals, which connect all polyps in a coral colony, exist only in the complex coral clade, undertaking the role of information transmission and material transportation among polyps. We believe that the changes in polyp-canal systems stem from growth needs in coral evolution. In contrast, the trend of coral evolution is toward the direction of higher complexity, more substantial growth consistency, and better niche occupation ability.

In the robust coral clade, the transition from solitary to compound corals necessitated the development of greater connection among polyps during growth and budding processes. The separated pores and connected budding sites fulfilled this demand (Fig. 2 and Fig. S3). The shift from large to small polyps led to a considerable increase in polyp quantity in the colony and the need for consistency in coral formation. Inter-septal spaces became the only type of lumen in *Pocillopora* and *Stylophora* corals, and their regular N type polyp-canal system prompted synergistic coral growth (Fig. S4). With this system, polyps in these genera exist in adjacent inter-septal spaces at the coral surface, growing, budding, and forming calices synchronously in the form of growth rings along their growth axis [20] (Fig. S4C).

To reach higher linear extension rates in the complex coral clade, polyps maintained their connections at a larger distance in their colonies through more refined polyp-canal systems (Figs. 3 to 5 and Figs. S6 to S11). Such canal networks comprise multiple canal types with different morphology, distributions, and functions to meet the requirements for more consistent growth in *Montipora* and *Acropora* colonies. This consistency is reflected in calices with similar volume (Fig. 7) and the synchronous polyp growth along the growth axes [13]. Meanwhile, the regulation of growth by the canal networks in *Montipora* and *Acropora* prevents the death of polyps due to inconsistent growth rates, such as that observed in *Galaxea* (Fig. S6C).

Due to the absence of mesh-like canals, the evolution of structural complexity in robust corals is slower, which also limits the ability of their communities to expand substantially and occupy niches. The emergence of mesh-like canals led to an evolutionary gap between robust and complex coral clades, and the evolution rate of structural complexity was greatly increased. Moreover, complex canal networks appeared in *Montipora* canals, and corals’ growth rate and community expansion ability were greatly increased. As for the *Acropora* corals, the appearance of axial canals increases the complexity of coral structure and the consistency of polyp growth to an unprecedented level, while *M. stellata* seems to be the transition species of this leap. It makes *Acropora* species with the most substantial growth and ecological occupation ability, with axial niche expansion and fragmentation dispersion patterns.

Interestingly, the evolution of polyp-canal systems is similar to the hierarchy from the cell to organ level (Fig. 1). The polyp of a solitary coral is analogous to a cell, while the polyp-canal system in compound corals is reminiscent of a group of cells. N type and P type systems are like loose cell masses without tight cell–cell connections, while the polyps in the M type system are closely related and arranged into tissues. Just like different tissues are further arranged in an orderly manner to form an organ, the C-2 type system in *Montipora* corals and the C-3 type system in *Acropora* corals consist of various canal types with different functions in deterministic forms, allowing for fine detail in skeletal development. Thus, the emergence of canal networks integrated the polyp-canal system into an entity very much like an organ system of a higher animal [14], promoting an increased rate of evolution in reef-building corals [26].

### Coral growth patterns affect their distribution in the habitat

The visualization of dynamic coral growth processes in this work reveals that coral growth patterns, including polyp budding directions, polyp-canal connections, and skeleton formation, can impact the distribution of reef-building corals in coral reefs.

*Acropora* corals dominate the reefs in a wide range of habitats [27–31]. Since the Quaternary Period (1.8 million years ago), this genus has reigned supreme in the low-nutrient and high-energy reef environments of the Indo-Pacific region [32–35]. This overwhelming evolutionary success appears to be based on the superiority of its C-3 type polyp-canal system. Specifically, the differences between canals (separated pores or mesh-like canals) and canal networks led to the evolution of coral growth patterns from “surfaces” to “lines” and further to “points” (Figs. 2 to 5).

The corals with P or M type systems and no canal network, like *Favia*, *Platygyra*, and *Porites*, can only indeterminately form their skeletons over the entire surface and occupy niches inefficiently (Figs. 2D and 3C and Fig. S3D). Meanwhile, *Montipora*, another coral genus with a canal network in the *Acroporidae* family, accounts for about 70% of the total coral coverage on the inner reef flat [36,37]. With their C-2 type system, the canal network synergizes adjacent polyps into an organized whole, and the calices form with similar shapes and sizes (Fig. S15). After their own calices are formed to the required size, most of the precursor components for skeletal biomineralization secreted by these polyps (e.g., inorganic ions and skeletal organic matrix) are transported to the colony edge through the tubular canals below the polyps [38–41] (Fig. 1). Skeleton formation then occurs at a high rate along the line of the colony edge. Laminar *Montipora* colonies displaying whorl-like growth can rapidly expand laterally, occupying most flat habitats in coral reefs [42] (Fig. 4B). The advent of the axial canal resulted in more complex and versatile canal networks in *Acropora* corals. The canal networks in axial and radial corallites facilitate highly deterministic growth forms for coral polyps and skeletons [14]. The calices are also formed into similar sizes, and the high rate of calcification at the branch tip is maintained by the translocation of products from all polyps in the branch via the gastrovascular system. The skeleton formation is mainly focused on the point of the branch tip, along the direction of the axial canal (Fig. 5B). High rates of calcification are secondary to the high rates of colony extension along the desired direction, ensuring the rapid framework growth of *Acropora* colonies in low-nutrient and high-energy habitats [27,32,42]. In addition, the axial canal is mainly involved in the mechanism of temporary calcium storage and redistribution in new branch-forming and colony self-healing processes, ensuring very rapid growth and local dispersion through fragmentation of *Acropora* corals [31,43,44]. With these characteristics, *Acropora* species can grow more rapidly and exploit a broader range of habitats in the Indo-Pacific region. In summary, accompanied by the evolution of polyp-canal connections and the emergence of canal networks, coral growth patterns became increasingly efficient.

On the other hand, *Acropora* corals are susceptible to both biotic and abiotic stressors [45,46]. Highly developed polyp-canal systems and small corallites render these corals sensitive to heat shock, acidification, poor water quality, disease, and predator outbreaks. However, environmentally stressful habitats favor the growth of large, encrusting plocoid or cerioid colonies; for example, *Favia* and *Platygyra* are dominant in shallow-water subtidal habitats [47], *Porites* is dominant in back-reef or lagoonal habitats [48], and *Pocillopora* and *Stylophora* are the 2 dominant genera in frequently disturbed environments and can be found in almost all coral reef habitats [49,50]. Their large, dense, and hard skeletons effectively protect polyps subjected to these stressors. The transmission of coral diseases and bleaching is also reduced in N or P type systems (Fig. 1). Therefore, the evolution of the polyp-canal system facilitates the rational distribution of reef-building corals in coral reef ecosystems and further increases species richness and diversity in reef habitats. These dominant coral genera maximize the use of niches in their own specific habitats, which may explain why coral reefs can become the cornerstone of marine ecosystems despite covering less than 0.1% of the ocean [51,52].

In addition, we found that solitary representatives of both clades have potential to form “multiple canals” in early stages (Figs. S1 and S5), which may indicate that both clades have potential to form mesh-like canals, but because of different evolutionary and developmental trajectories, this skeleton-forming pattern is only fully expressed in colonial complex corals.

### Polyp and canal reconstructions visualize dynamic coral growth patterns

In this work, HRCT captured large quantities of structural data from the samples, and the 3D reconstructions we created simulated the growth-budding process of coral polyps. As there are hundreds of polyps in one small colony, the structural information of these reconstructions can effectively summarize the morphological characteristics at different stages in polyp growth and skeleton formation. Hence, we can visualize the changes in polyp size, form, position, connection, and surrounding canals during growth, budding, and other processes. Thus, it provides a valuable tool for studying the dynamic growth processes of reef-building corals from a morphological perspective.

The method used in this work not only is suitable for the study of coral evolution in robust and complex coral clades and their polyp-canal connections but also can support related coral studies, such as growth direction [14,21], calcium transport patterns [24], and reef rubble formation [25]. Furthermore, in future studies, this method can be used to explore polyp-canal systems in different experimental contexts, such as heat stress, acid stress, damage and self-healing, diurnal rhythm, toxicology, and the relationship between coral reef biota and coral skeleton structures. By reconstructing polyp-canal systems, changes in structure and growth patterns in response to external stressors can be investigated.

In comparison, few existing morphological or biological criteria can resolve robust and complex clades [12,13,15,16]. At this stage, it is difficult for omics or molecular biology research to elucidate the changes in polyp-canal connection and skeleton formation during evolution. Meanwhile, comparing a single gene battery has limitations in reflecting changes during complex biological processes. Due to the challenges of coral evolutionary mechanisms, the evolutionary trees obtained from different genetic groups are inconsistent. Therefore, evolutionary research based on polyp-canal system reconstructions is unique in its ability to reveal coral evolution toward complexity, complementing the current gap in reef-building coral studies [12,13]. This study also draws greater attention to canal networks, which will be relevant for future studies on coral growth.

## Materials and Methods

### Sample collection

The *A. millepora*, *A. muricata*, *C. vaughani*, *L. hemprichii*, *M. capricornis*, *M. foliosa*, *P. damicornis*, *P. meandrina*, *P. minuta*, *P. verrucosa*, and *S. pistillata* samples (2018) were collected from the Xisha Islands (15°46′N to 17°08′N, 111°11′E to 112°54′E); the *A. digitifera*, *F. speciosa* (2008), and *M. turgescens* (2017) samples were collected from the Nansha Islands (3°35′N to 11°55′N, 109°30′E to 117°50′E); the *P. lutea*, *T. peltata*, and *B. europaea* samples (2017) were collected from the Weizhou Islands (20°54′N to 21°10′N, 109°00′E to 109°15′E); the *G. fascicularis*, and *M. stellata* samples (2017) were collected from the Gaven Reef (10°13′N, 114°12′E); the *A. myriophthalma* samples (2017) were collected from the Reed Tablemount (11°20′N, 116°50′E); the *P. sinensis* samples (2017) were collected from the Subi Reef (10°54′N, 114°03′E). All samples were found in shallow tropical reefs, from depths of 5 to 10 m, and the daily mean temperature was between 23.2 and 29.2 °C. The coral samples were kept whole and housed in our laboratory coral tank, where all conditions were simulated to reflect those of their habitat in the South China Sea. The harvesting and farming permit for reef-building corals can be found in the Supplementary Materials. These samples were kept in the tank for about 1 to 3 months before HRCT analysis.

### Coral culture system

The coral samples were temporarily cultured in a standard RedSea tank (redsea575, Red Sea Aquatics Ltd., London, UK) before and after HRCT, following the Berlin method. The temperature was kept at 25 °C, and the salinity (Red Sea Aquatics Ltd., London, UK) was 1.025. The culture system was maintained using Protein Skimmer (regal250s, Honya Co. Ltd., Shenzhen, China), a water chiller (tk1000, TECO Ltd., Taiwan, China), 3 coral lamps (AI, Red Sea Aquatics Ltd., London, UK), 2 wave devices (VorTech MP40, EcoTech Marine Ltd., Bethlehem, USA), and a calcium reactor (Calreact 200, Honya Co. Ltd., Shenzhen, China).

### HRCT

Coral samples from the South China Sea were analyzed using 3D models constructed with a 230-kV latest-generation x-ray microfocus computed tomography system [Phoenix v|tome|x m, General Electric (GE)] at Yinghua NDT, Shanghai, China (Table S2). 2D image reconstructions of each specimen from the scan slice matrices were assembled using GE proprietary software.

### 3D polyp and canal reconstructions

Slice data derived from the scans were then analyzed and manipulated using 3D reconstruction software. The reconstructions of the polyp-canal system were performed using VG Studio Max (v3.3.0). The 3D reconstructions were created following the method previously described [21]. Images of the reconstructions were exported from Mimics and VG Studio Max and finalized in Adobe Photoshop CC 2019 and Adobe Illustrator CC 2019.

### Simulation of dynamic coral growth processes

In this work, there were hundreds of polyps in even a small branchlet of our samples (except *C. vaughani*, *L. hemprichii*, and *B. europaea*). Thus, we can obtain the growth information of all these polyps through the 3D reconstruction, which includes polyps of different growth stages. After comparing and sorting, we can arrange these polyp reconstructions to simulate the polyp morphology and distribution changes during coral growth. This method allows the characterization of dynamic coral growth processes by detecting and reconstructing static samples.

### Ethics

This article does not involve experiments on cephalopods or higher animals. All coral sample collection and processing were performed according to the local laws governing the welfare of invertebrate animals.

## Data Availability

The HRCT data supporting this study’s findings are available in Figshare. The 180 links to our files can be found in the Supplementary Materials.

## Supplementary Materials

Supplementary 1 Harvesting and farming permit Figs. S1 to S15 Table S1
