# Supplementary material for: Polyp-Canal Reconstruction Reveals Evolution Toward Complexity in Corals
Source: Research (Wash D C). 2023 Jun 6;6:0166. doi: 10.34133/research.0166 (PMC10243894; doi:10.34133/research.0166)
Supplement: Supplementary 1 — Harvesting and farming permit Figs. S1 to S15 Table S1 [file research.0166.f1.zip › Harvesting and farming permit.pdf]

中 华 人 民 共 和 国
水生野生动物人工繁育许可证

（琼 ）水野繁育字（2022 ）001 号

单位(个人)：海南省海洋与渔业科学院

地　　址：海南省海口市美丽区演丰镇演海村委会石路村北侧

法 人 代 表：王道儒

物 种 学 名：国家重点保护、《公约》附录物种（详见副本）

有 效 期 至：2025-01-05

发证机关：海南省农业农村厅

（公章）

2022年 1 月 5 日

海南省农业农村厅 行政审批专用章

农业农村部渔业渔政管理局制

中华人民共和国水生野生动物

人 工 繁 育 许 可 证

（副　　本）

编号:（琼　）水野繁育字［2022］ 001号

单位(个人)名称：海南省海洋与渔业科学院

地　　　址：海南省海口市美丽区演丰镇演海村委会石路村北侧

法 人 代 表：王道儒

技术负责人：吴钟解

人工繁育目的：科学研究

有 效 期 至：2025-01-05

发证机关：海南省农业农村厅

（盖章）

2022年 1 月 5 日

农业农村部渔业渔政管理局制

持 证 须 知

1、持证单位（个人）在证件有效期内享有本证所列水生野生动物的合法人工繁育权利。

2、持证单位（个人）必须遵守《中华人民共和国野生动物保护法》、《中华人民共和国水生野生动物保护实施条列》等有关法律法规的规定。

3、持证单位（个人）须详细记录许可物种的人工繁育情况，并接受各级渔业行政主管部门监督检查。

4、本证每年至少审核一次，未经审核，本证无效。

5、本证只供本单位（个人）使用，不得伪造、涂改、转让或者买卖。

6、本证应妥善保存，发现遗失、严重损坏，须立即报告发证机关。

年度检验情况			

人工繁育物种情况

物种学名	保护级别		来源	数量及变更情况
	CITES	国家		
鹿角杯形珊瑚 *Pocillopora damicornis*	II级	二级	捕捉-（琼）水野捕字（2021）001号	1株
疣状杯形珊瑚 *Pocillopora verrucose*	II级	二级	捕捉-（琼）水野捕字（2021）001号	1株
多曲杯形珊瑚 *Pocillopora meandrina*	II级	二级	捕捉-（琼）水野捕字（2021）001号	1株
多孔鹿角珊瑚 *Acropora millepora*	II级	二级	捕捉-（琼）水野捕字（2021）001号	1株
芽枝鹿角珊瑚 *Acropora gemmifera*	II级	二级	捕捉-（琼）水野捕字（2021）001号	1株
风信子鹿角珊瑚 *Acropora hyacinthus*	II级	二级	捕捉-（琼）水野捕字（2021）001号	1株
强壮鹿角珊瑚 *Acropora valida*	II级	二级	捕捉-（琼）水野捕字（2021）001号	1株

人工繁育物种情况

物种学名	保护级别		来源	数量及变更情况
	CITES	国家		
粗野鹿角珊瑚 *Acropora humilis*	II级		捕捉-（琼）水野捕字（2021）001号	1株
丘突鹿角珊瑚 *Acropora abrotanoides*	II级	二级	捕捉-（琼）水野捕字（2021）001号	1株
叶状蔷薇珊瑚 *Montipora foliosa*			捕捉-（琼）水野捕字（2021）001号	1株
指状蔷薇珊瑚 *Montipora digitata*	II级	二级	捕捉-（琼）水野捕字（2021）001号	1株
细枝鹿角珊瑚 *Acropora nana*	II级	二级	捕捉-（琼）水野捕字（2021）001号	1株
多星孔珊瑚 *Astreopora myriophthalma*	II级	二级	捕捉-（琼）水野捕字（2021）001号	1株
辐石芝珊瑚 *Heliofungia actiniformis*	II级		捕捉-（琼）水野捕字（2021）001号	1株

人工繁育物种情况

物种学名	保护级别		来源	数量及变更情况
	CITES	国家		
绕石珊瑚 *Herpolitha limax*	II级	二级	捕捉-（琼）水野捕字（2021）001号	1株
膨胀蔷薇珊瑚 *Montipora turgescens*	II级	二级	捕捉-（琼）水野捕字（2021）001号	1株
瘿叶蔷薇珊瑚 *Montipora aequituberculata*	II级	二级	捕捉-（琼）水野捕字（2021）001号	1株
厚板牡丹珊瑚 *Pavona duerdeni*	II级	二级	捕捉-（琼）水野捕字（2021）001号	1株
标准厚丝珊瑚 *Pachyseris speciosa*	II级	二级	捕捉-（琼）水野捕字（2021）001号	1株
皱纹厚丝珊瑚 *Pachyseris rugosa*	II级	二级	捕捉-（琼）水野捕字（2021）001号	1株
澄黄滨珊瑚 *Porites lutea*	II级	二级	捕捉-（琼）水野捕字（2021）001号	1株

人工繁育物种情况

物种学名	保护级别		来源	数量及变更情况
	CITES	国家		
团块滨珊瑚 *Porites lobata*	II级	二级	捕捉-（琼）水野捕字（2021）001号	1株
地衣滨珊瑚 *Porites lichen*	II级	二级	捕捉-（琼）水野捕字（2021）001号	1株
普哥滨珊瑚 *Porites pukoensis*			捕捉-（琼）水野捕字（2021）001号	1株
平滑穴孔珊瑚 *Alveopora tizardi*	II级	二级	捕捉-（琼）水野捕字（2021）001号	1株
丛生盔形珊瑚 *Galaxea fascicularis*	II级	二级	捕捉-（琼）水野捕字（2021）001号	1株
秘密角蜂巢珊瑚 *Favites abdita*	II级	二级	捕捉-（琼）水野捕字（2021）001号	1株
梳状菊花珊瑚 *Goniastrea pectinata*	II级	二级	捕捉-（琼）水野捕字（2021）001号	1株

海南省农业农村厅 行政审批专用章

人工繁育物种情况

物种学名	保护级别		来源	数量及变更情况
	CITES	国家		
中华扁脑珊瑚 *Platygyra sinensis*	II级	二级	捕捉-（琼）水野捕字（2021）001号	1株
精巧扁脑珊瑚 *Platygyra daedalea*	II级	二级	捕捉-（琼）水野捕字（2021）001号	1株
小扁脑珊瑚 *Platygyra pini*	II级	二级	捕捉-（琼）水野捕字（2021）001号	1株
带刺蜂巢珊瑚 *Favites stelligera*	I级	二级	捕捉-（琼）水野捕字（2021）001号	1株
大圆菊珊瑚 *montastrea magnistellata*	I级	二级	捕捉-（琼）水野捕字（2021）001号	1株
板叶角蜂巢珊瑚 *Favites complanata*	II级	二级	捕捉-（琼）水野捕字（2021）001号	1株
宝石刺孔珊瑚 *Echinopora gemmacea*	II级	二级	捕捉-（琼）水野捕字（2021）001号	1株

海南省农业农村厅行政审批专用章

人工繁育物种情况

物种学名	保护级别		来源	数量及变更情况
	CITES	国家		
扁枝滨珊瑚 *Porites andrewsi*	I级	二级	捕捉-（琼）水野捕字（2021）001号	1株

人工繁育物种情况

物种学名	保护级别		来源	数量及变更情况
	CITES	国家		

人工繁育物种情况

物种学名	保护级别		来源	数量及变更情况
	CITES	国家		
