# Supplementary figures and images for: Polyp-Canal Reconstruction Reveals Evolution Toward Complexity in Corals
Source: Research (Wash D C). 2023 Jun 6;6:0166. doi: 10.34133/research.0166 (PMC10243894; doi:10.34133/research.0166)

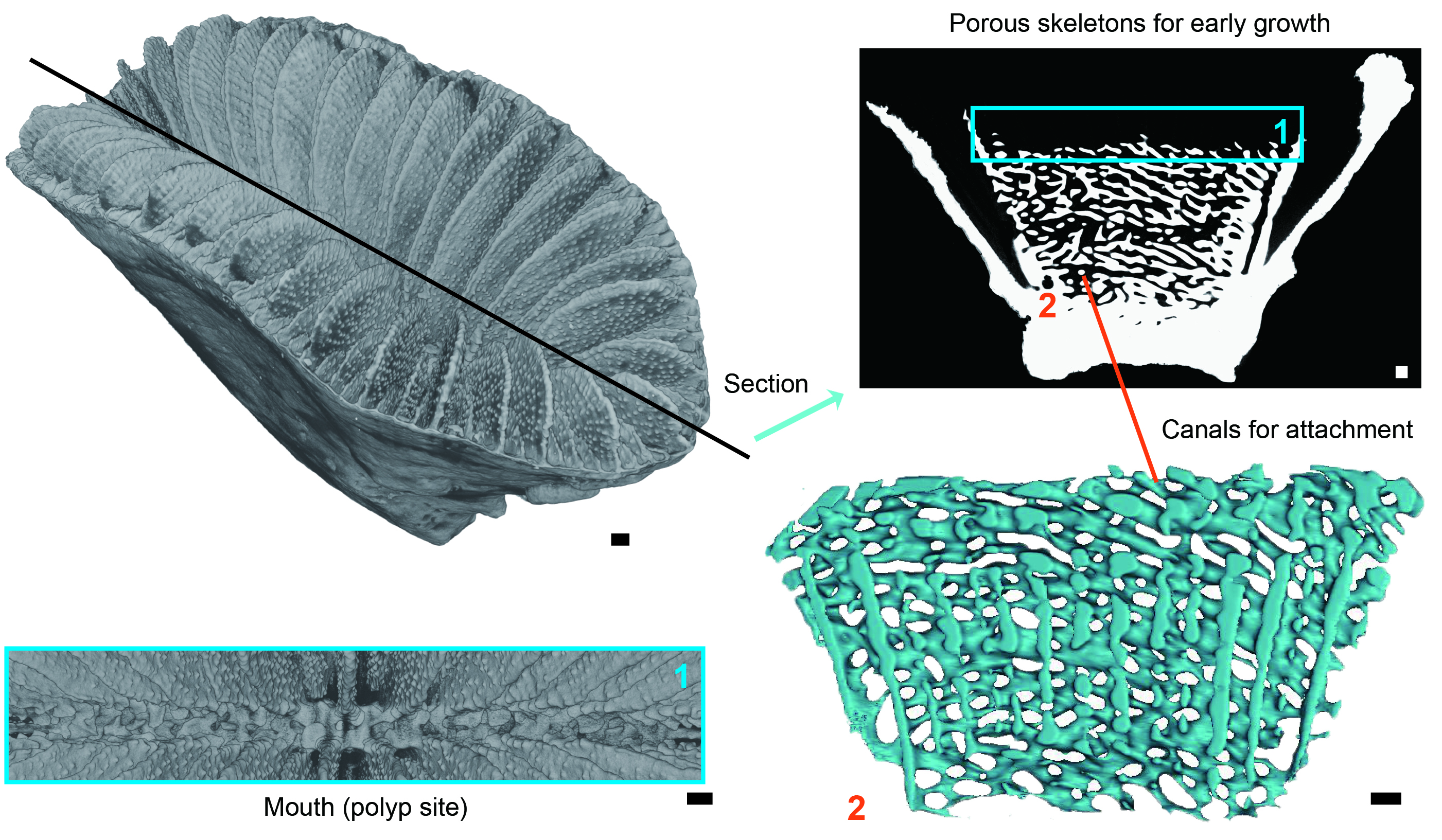

Supplement: Supplementary 1 — Harvesting and farming permit Figs. S1 to S15 Table S1 [file research.0166.f1.zip › Figure S5.jpg]

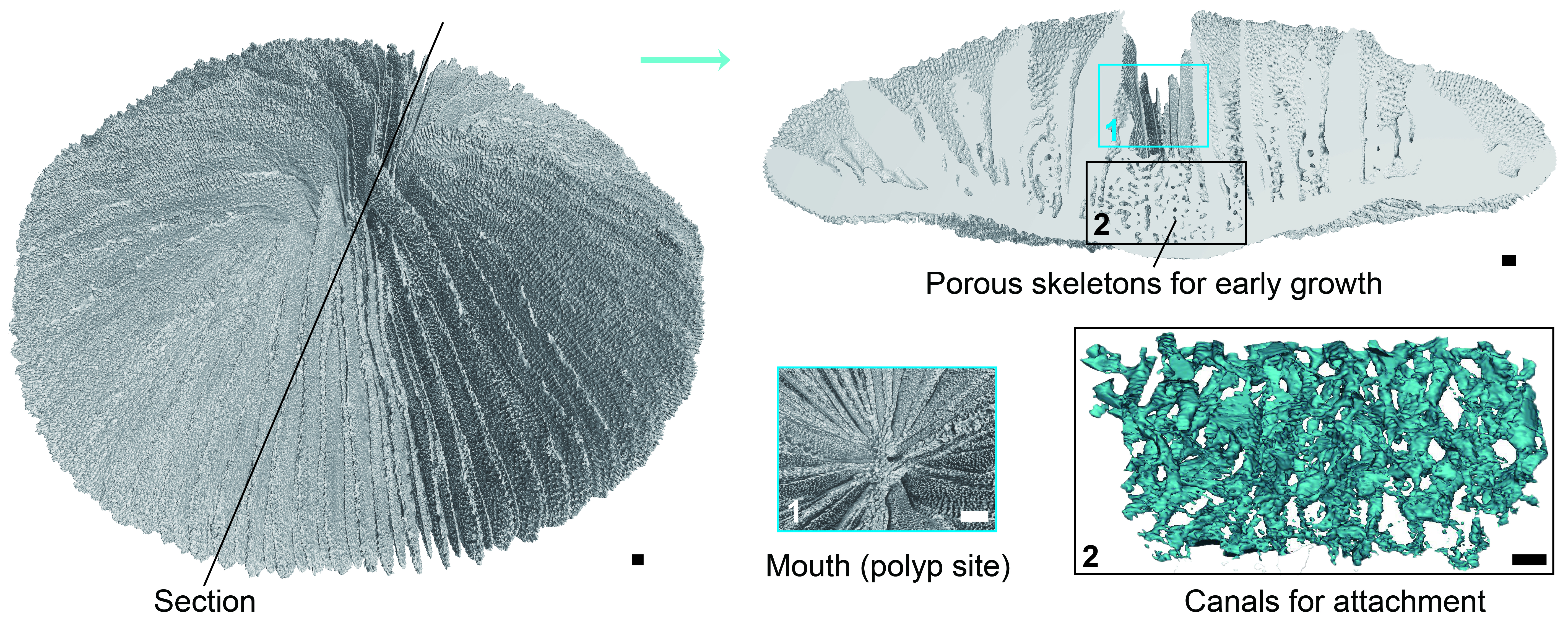

Supplement: Supplementary 1 — Harvesting and farming permit Figs. S1 to S15 Table S1 [file research.0166.f1.zip › Supplementary Figure 1.jpg]

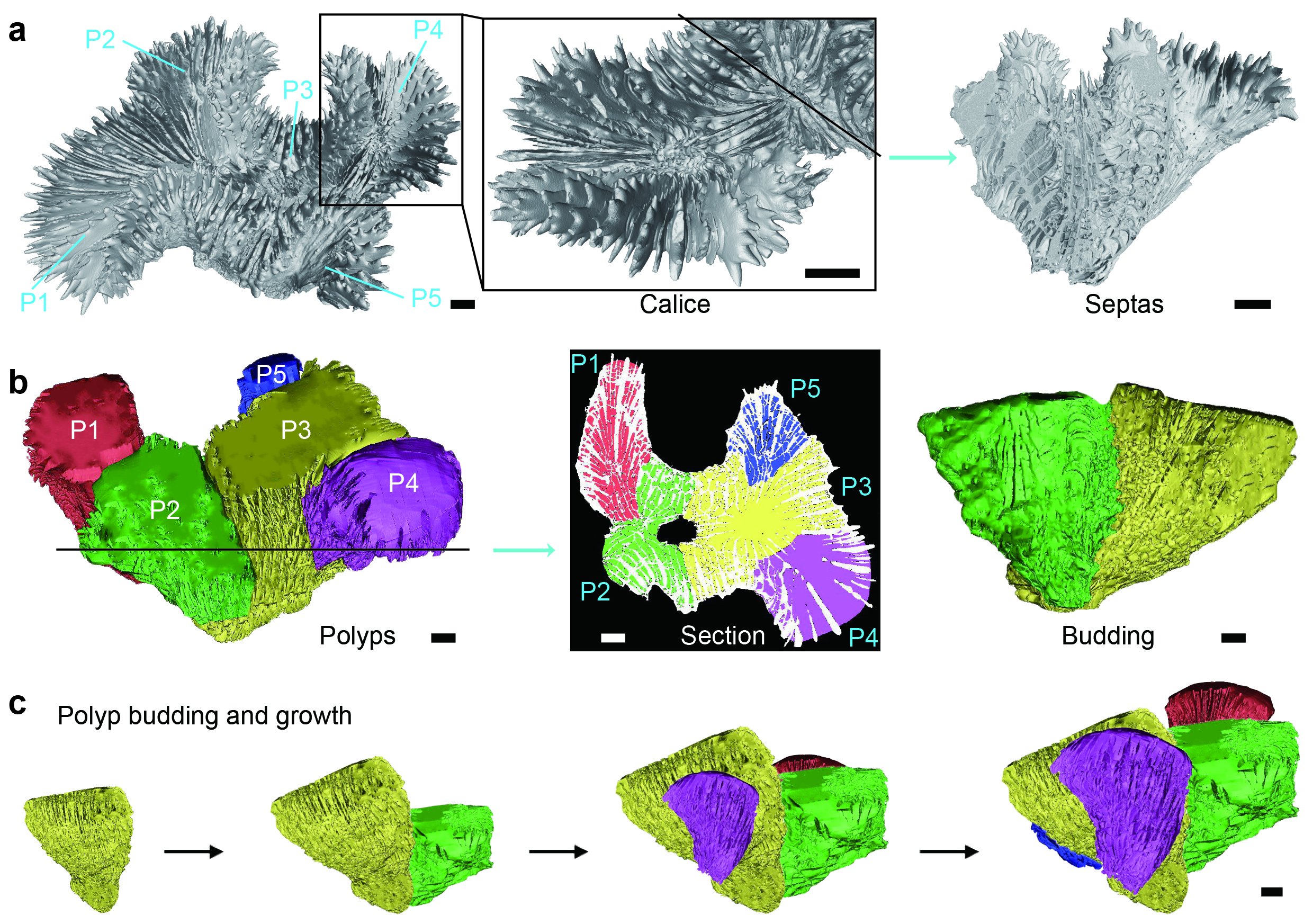

Supplement: Supplementary 1 — Harvesting and farming permit Figs. S1 to S15 Table S1 [file research.0166.f1.zip › Supplementary Figure 2.jpg]

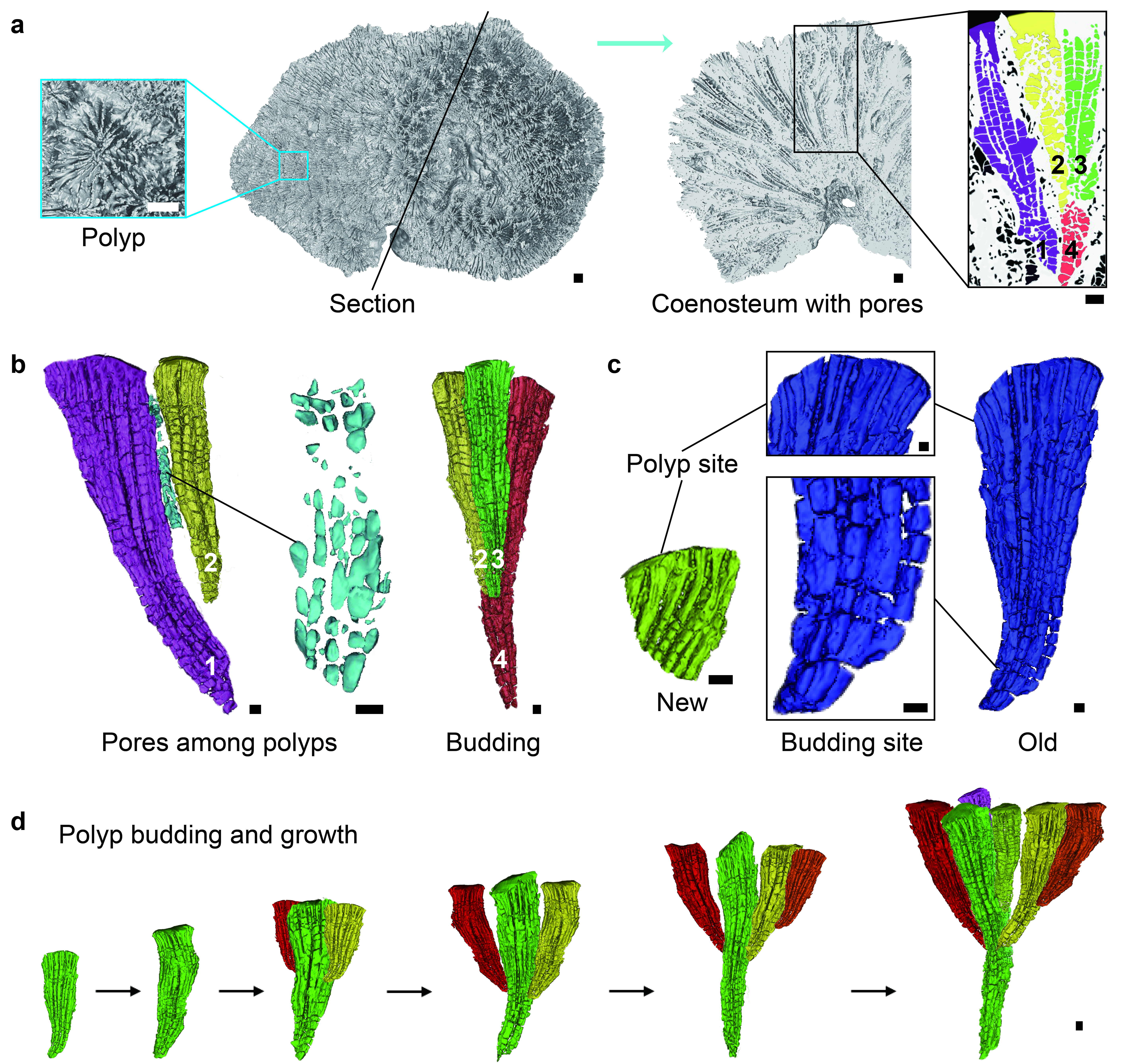

Supplement: Supplementary 1 — Harvesting and farming permit Figs. S1 to S15 Table S1 [file research.0166.f1.zip › Supplementary Figure 3.jpg]

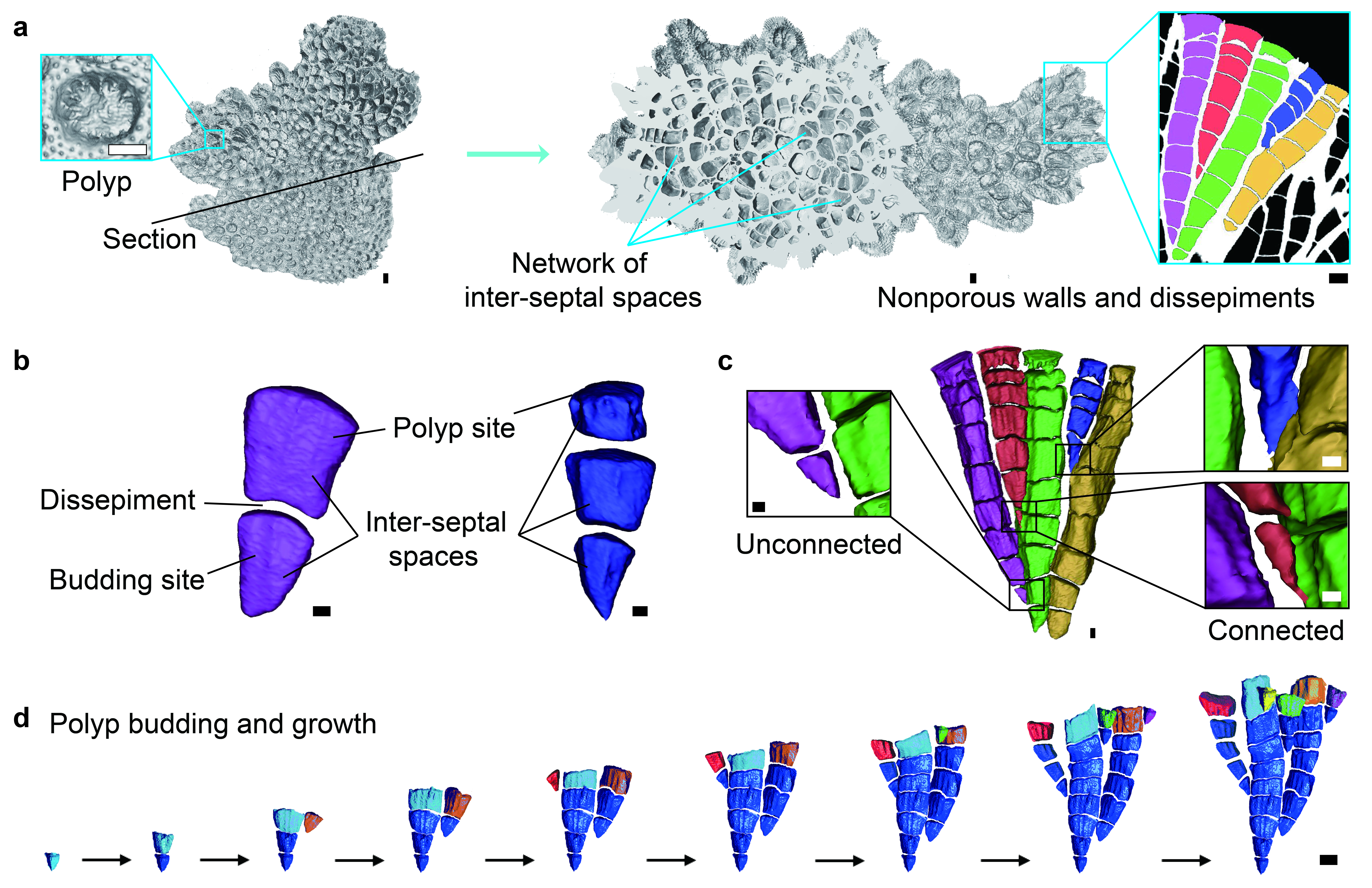

Supplement: Supplementary 1 — Harvesting and farming permit Figs. S1 to S15 Table S1 [file research.0166.f1.zip › Supplementary Figure 4.jpg]

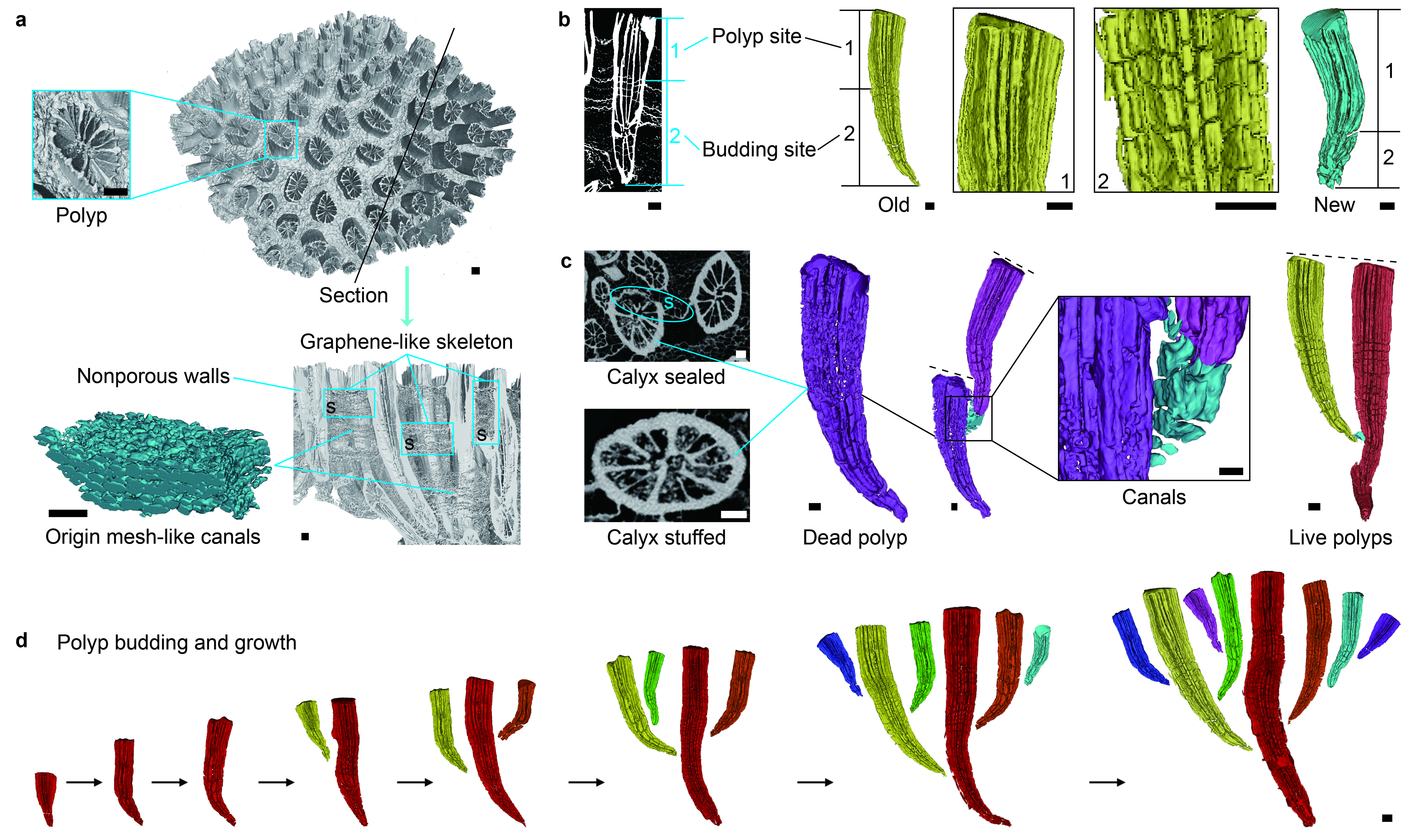

Supplement: Supplementary 1 — Harvesting and farming permit Figs. S1 to S15 Table S1 [file research.0166.f1.zip › Supplementary Figure 6.jpg]

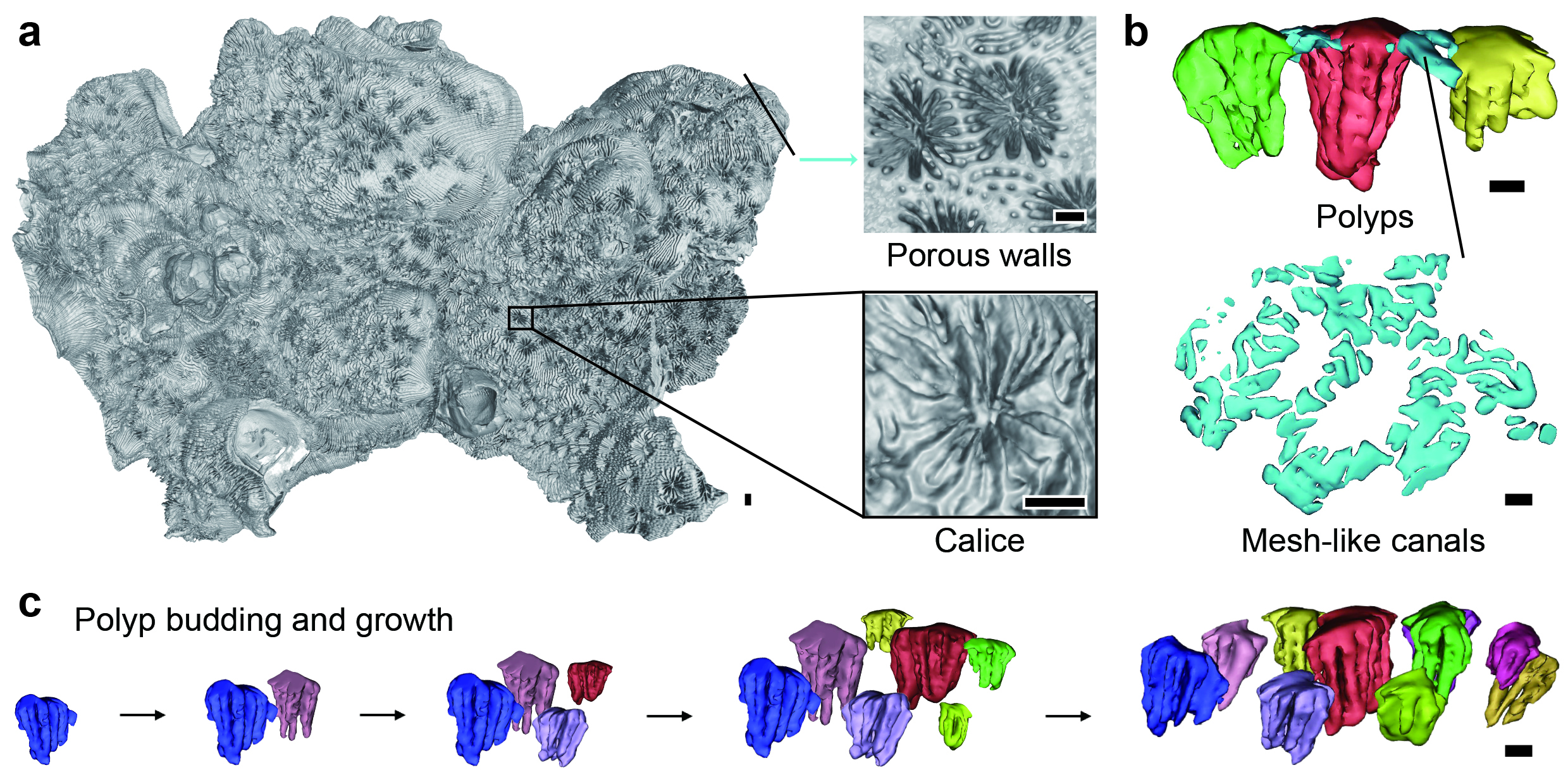

Supplement: Supplementary 1 — Harvesting and farming permit Figs. S1 to S15 Table S1 [file research.0166.f1.zip › Supplementary Figure 7.jpg]

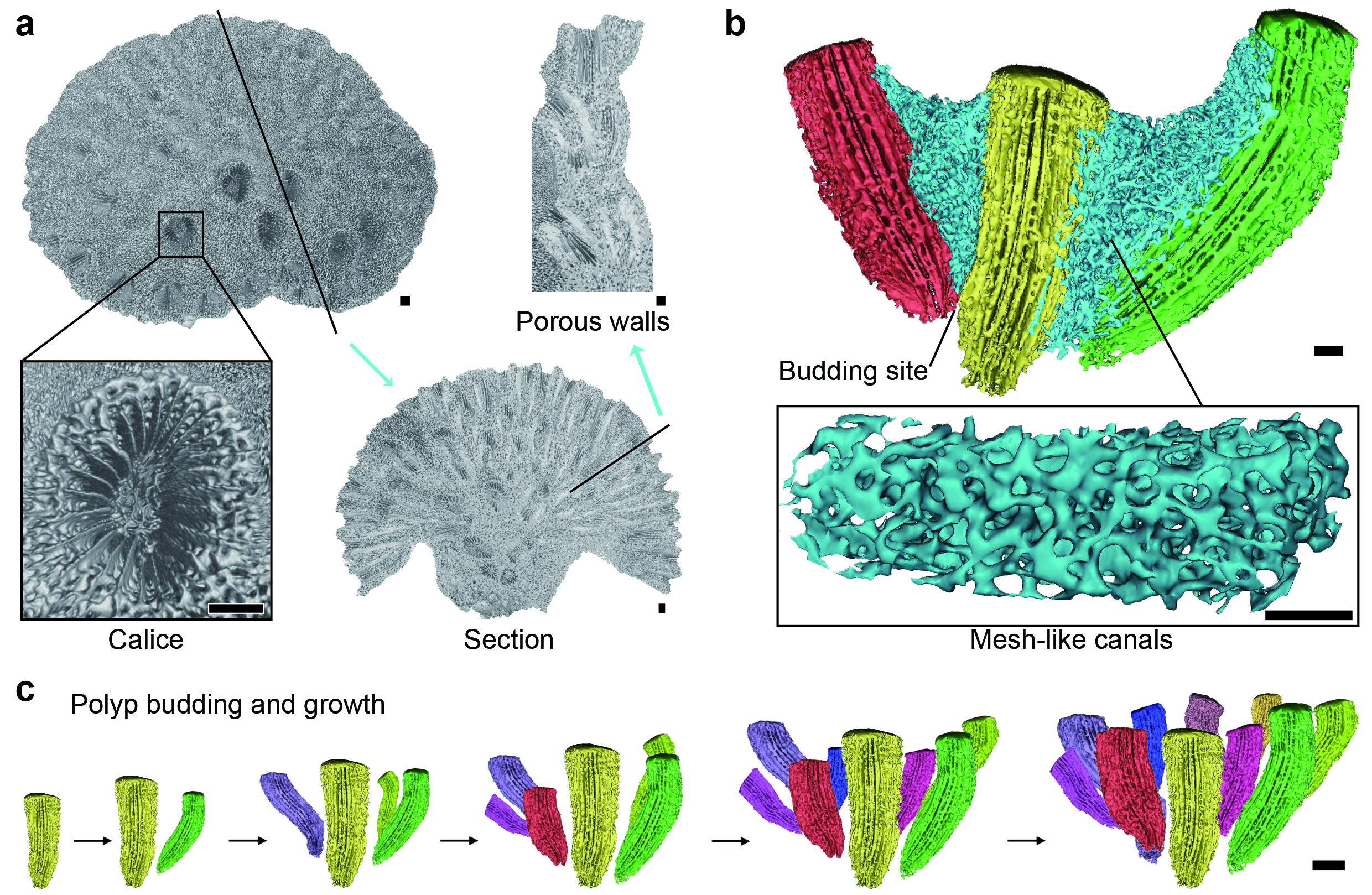

Supplement: Supplementary 1 — Harvesting and farming permit Figs. S1 to S15 Table S1 [file research.0166.f1.zip › Supplementary Figure 8.jpg]

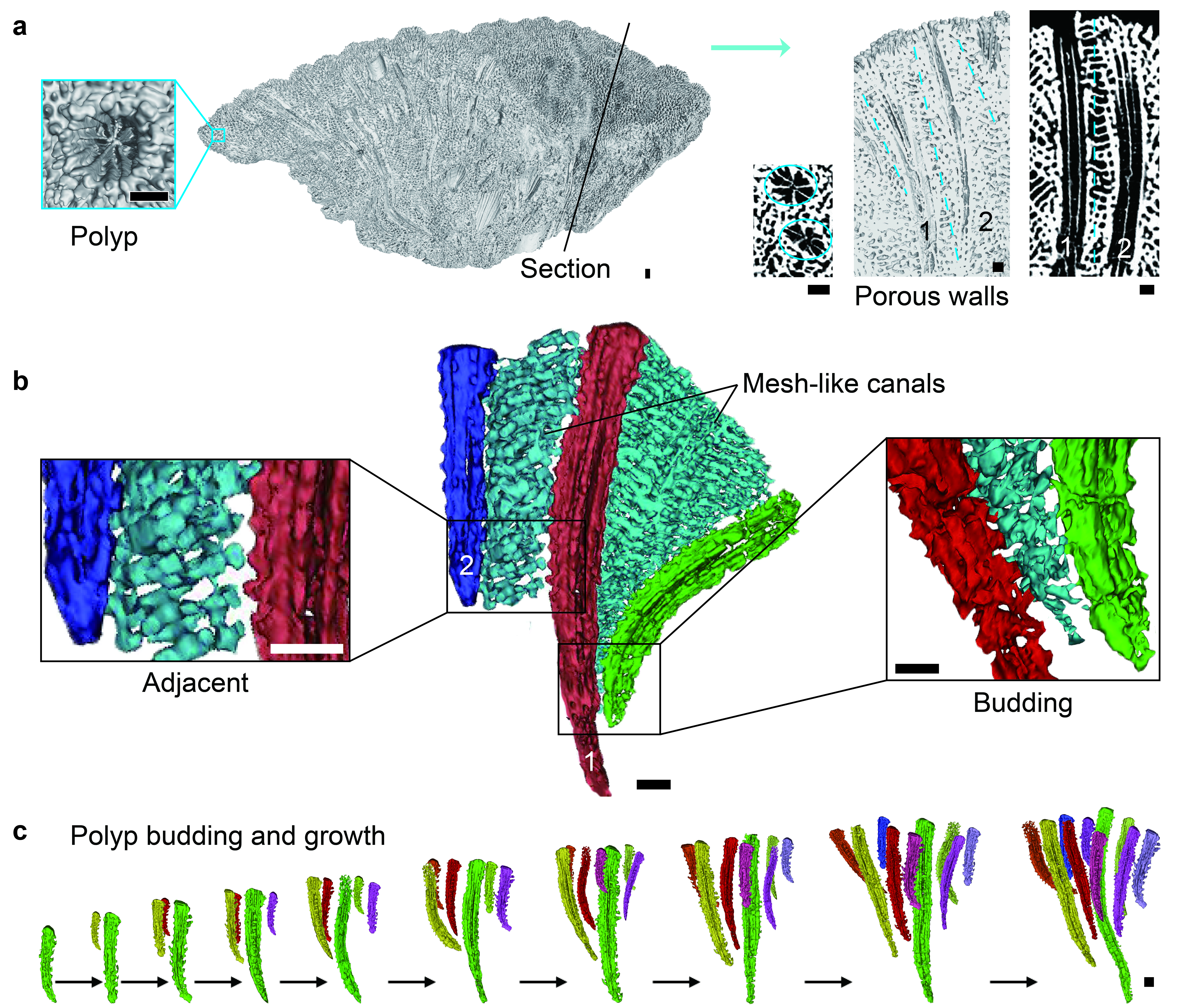

Supplement: Supplementary 1 — Harvesting and farming permit Figs. S1 to S15 Table S1 [file research.0166.f1.zip › Supplementary Figure 9.jpg]

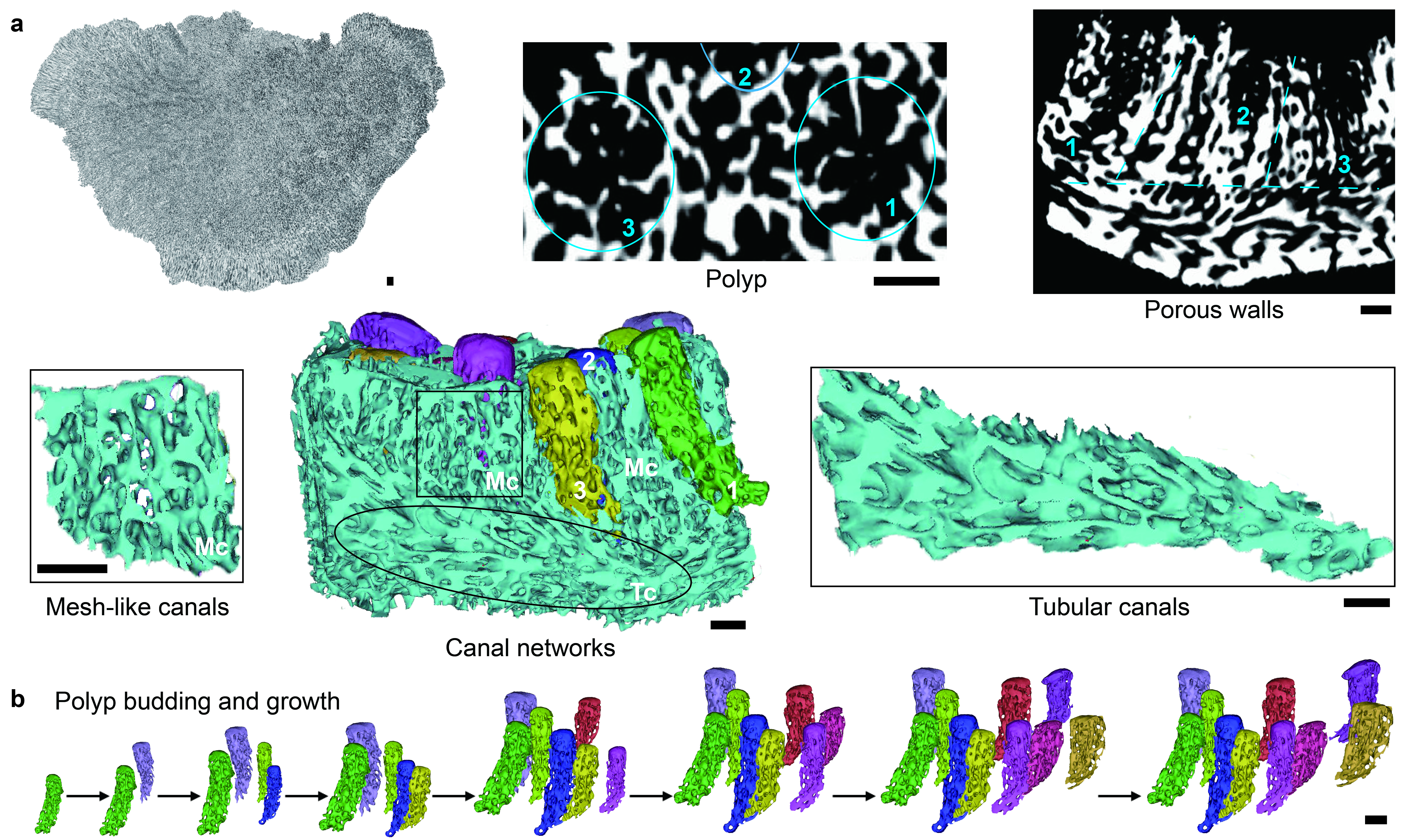

Supplement: Supplementary 1 — Harvesting and farming permit Figs. S1 to S15 Table S1 [file research.0166.f1.zip › Supplementary Figure 10.jpg]

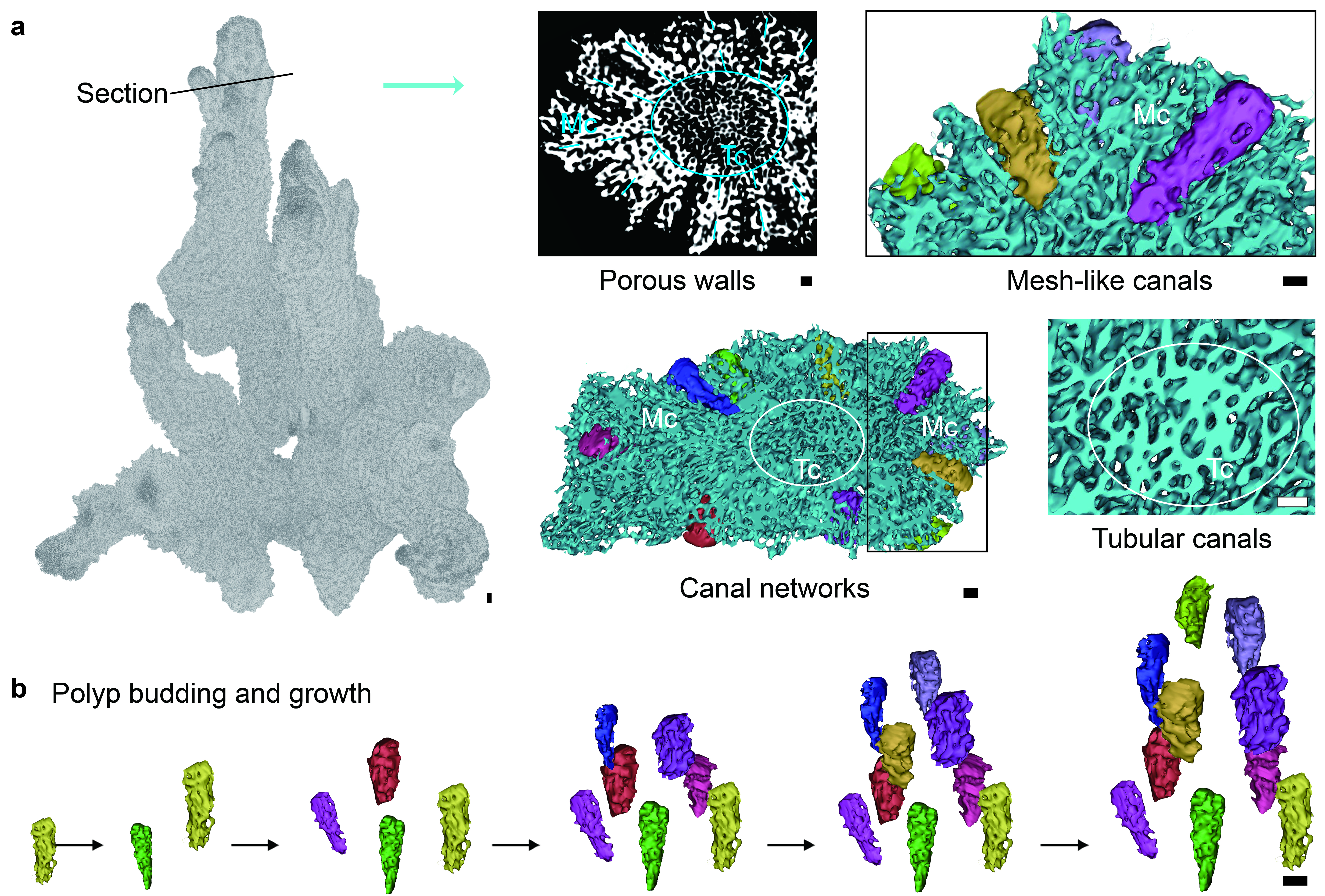

Supplement: Supplementary 1 — Harvesting and farming permit Figs. S1 to S15 Table S1 [file research.0166.f1.zip › Supplementary Figure 11.jpg]

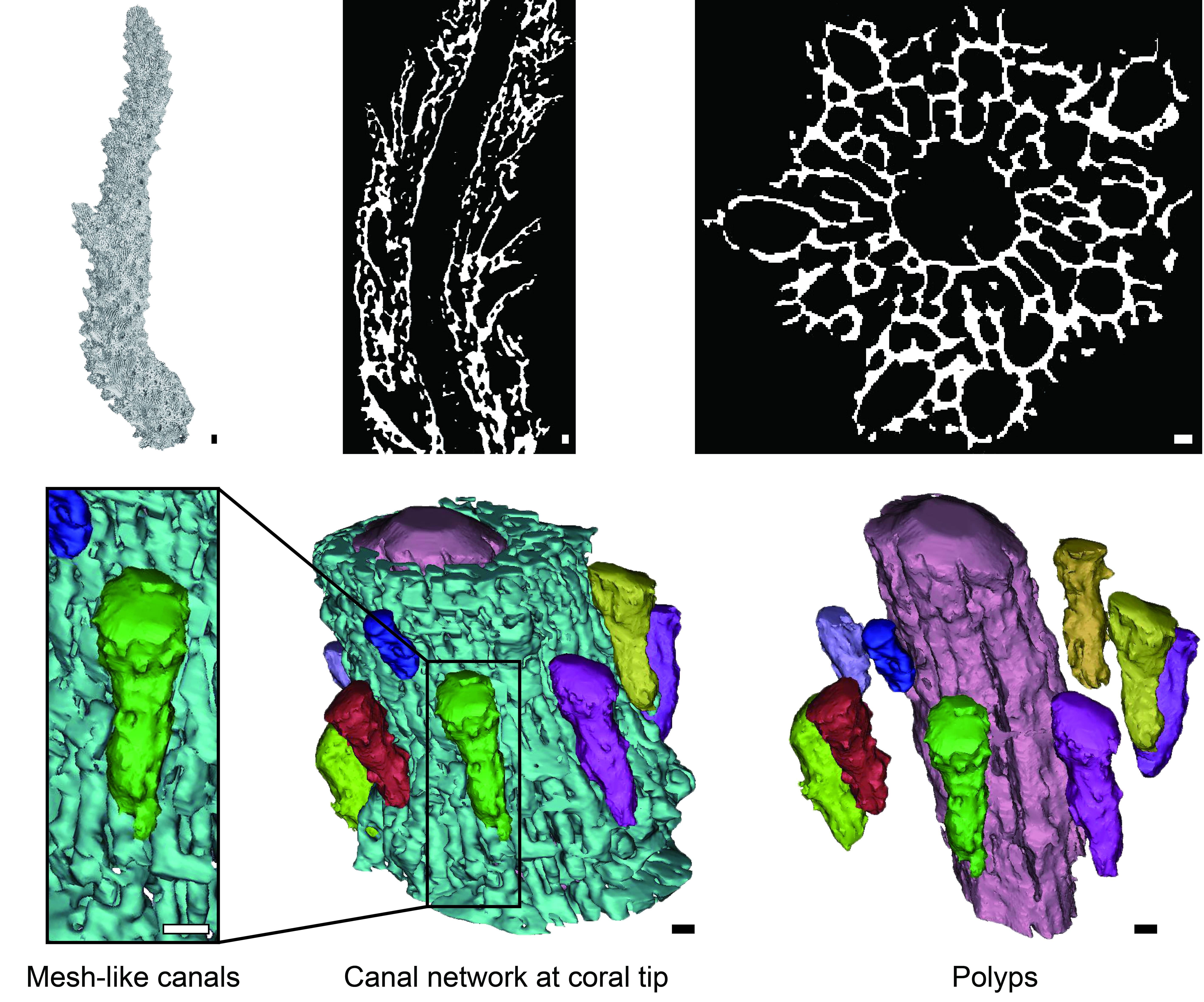

Supplement: Supplementary 1 — Harvesting and farming permit Figs. S1 to S15 Table S1 [file research.0166.f1.zip › Supplementary Figure 12.jpg]

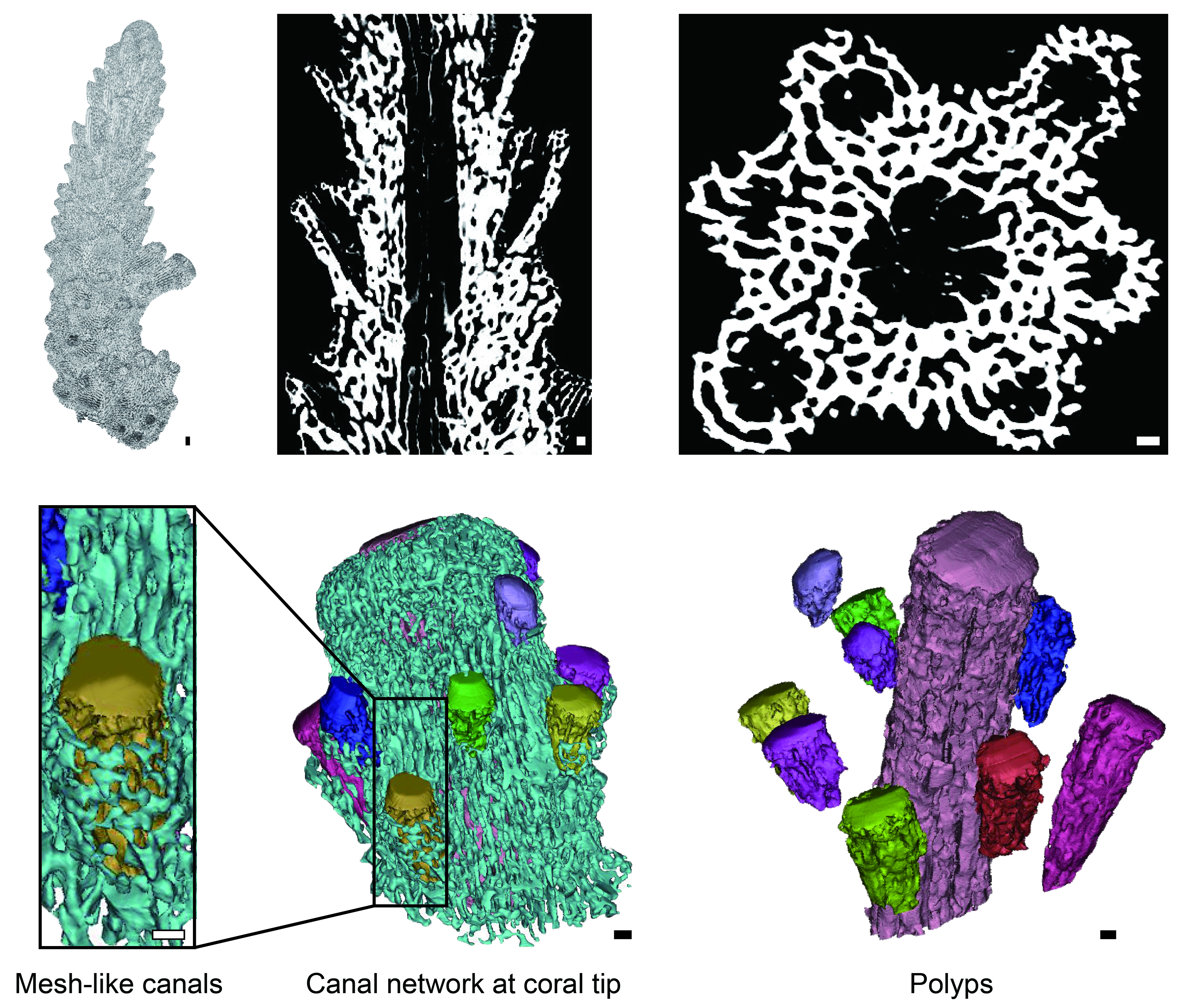

Supplement: Supplementary 1 — Harvesting and farming permit Figs. S1 to S15 Table S1 [file research.0166.f1.zip › Supplementary Figure 13.jpg]

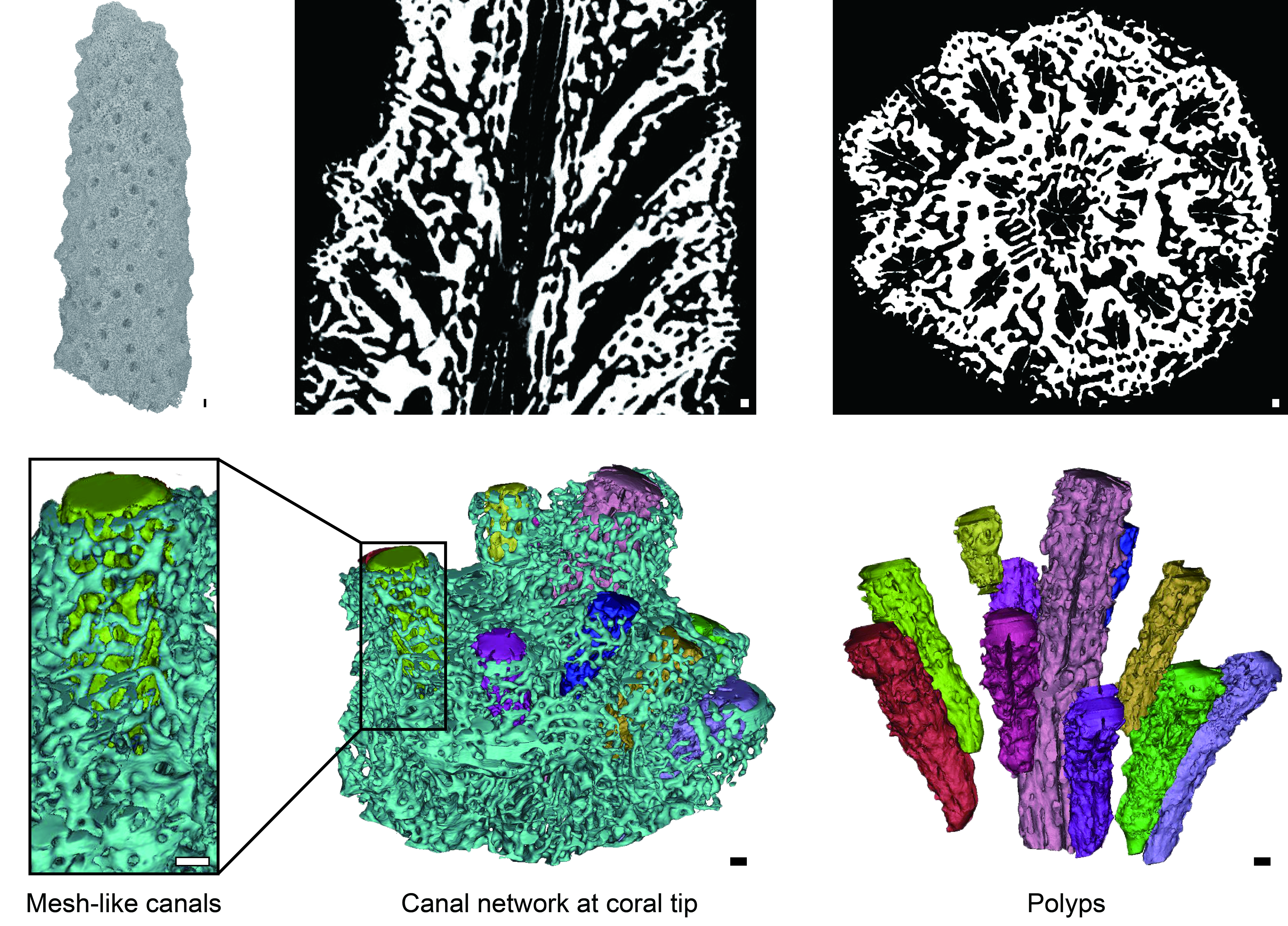

Supplement: Supplementary 1 — Harvesting and farming permit Figs. S1 to S15 Table S1 [file research.0166.f1.zip › Supplementary Figure 14.jpg]

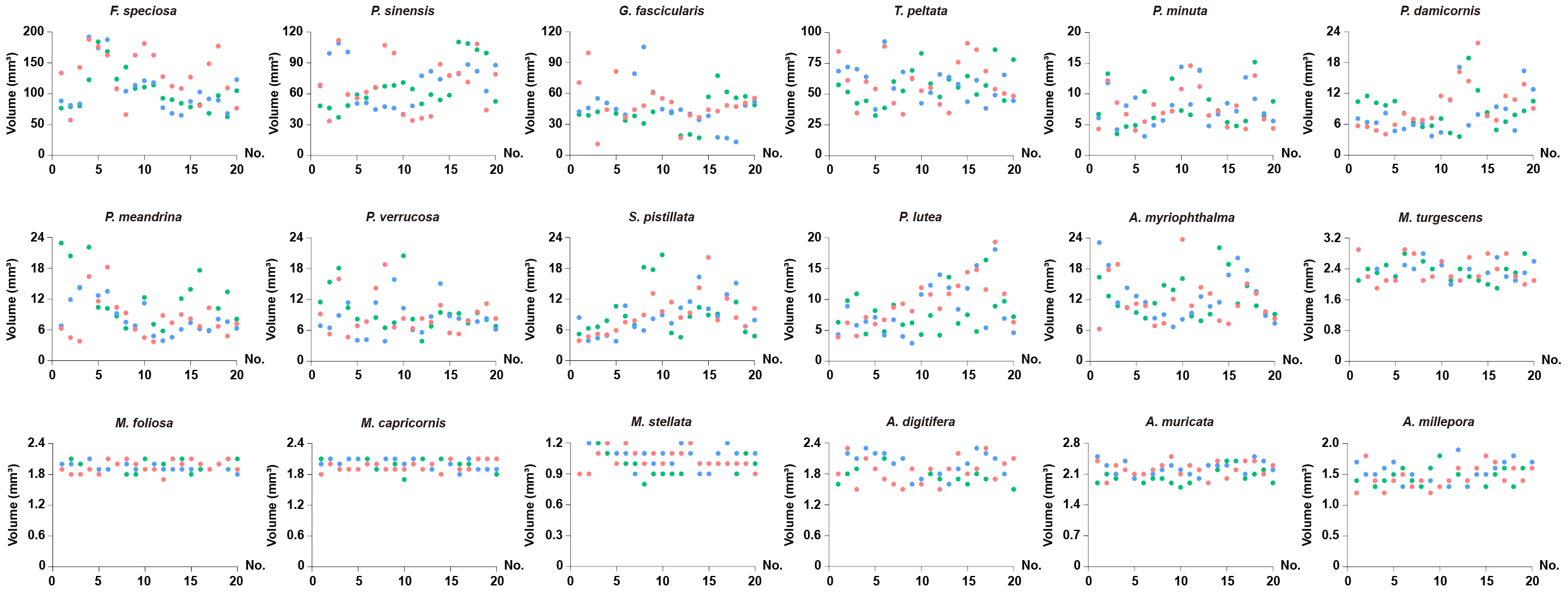

Supplement: Supplementary 1 — Harvesting and farming permit Figs. S1 to S15 Table S1 [file research.0166.f1.zip › Supplementary Figure 15.jpg]
